# Engineering *Saccharomyces cerevisiae* to improve heterologous abscisic acid production

**DOI:** 10.1186/s12934-025-02913-8

**Published:** 2026-01-23

**Authors:** Maximilian Otto, Sara Muñiz-Calvo, Michael Gossing, Florian David, Verena Siewers

**Affiliations:** 1 Division of Systems and Synthetic Biology, Department of Life Sciences, Chalmers University of Technology, Gothenburg, Sweden; 2 Discovery Sciences, Biopharmaceuticals R&D, AstraZeneca, Gothenburg, Sweden; 3 Novo Nordisk Foundation Center for Biosustainability, Technical University of Denmark, Kgs. Lyngby, Denmark

**Keywords:** *Saccharomyces cerevisiae*, *Botrytis cinerea*, Metabolic engineering, Abscisic acid, Cytochrome P450 monooxygenases, Endoplasmic reticulum, *PAH1*, *AtMSBP1*, *AtCOL4*

## Abstract

**Background:**

Abscisic acid (ABA) is a phytohormone involved in regulating plant growth, development, and stress responses. Its various physiological activities in plants and animals make the molecule a high-value product with agricultural, medical and nutritional applications. We previously constructed an ABA cell factory by expressing the ABA metabolic pathway from *Botrytis cinerea* in the biotechnological workhorse *Saccharomyces cerevisiae*. In this study, we aimed to improve ABA production and explored various rational engineering targets mostly focusing on increasing the activity of the two cytochrome P450 monooxygenases of the ABA pathway, BcABA1 and BcABA2. We evaluated the effects of cell membrane transporters, expression of heterologous cytochrome b5, improving heme supply, altering ER homeostasis, expression of *Arabidopsis thaliana* proteins and improving the precursor supply.

**Results:**

One of the genes involved in ER membrane homeostasis, *PAH1*, was identified as a promising engineering target. Knock-out of *PAH1* improved ABA titers but also caused a severe growth defect. By replacing the *PAH1* promoter with a weak minimal promoter, it was possible to mediate the growth defect while still improving ABA production. However, we also found that, in terms of ABA titer, a strain expressing the *A. thaliana* genes encoding the membrane steroid binding protein 1 (*AtMSBP1*) and the putative transcription factor CONSTANS-like 4 protein (*AtCOL4*) outperformed a highly-engineered strain with two copies of *bcaba1* and *bcaba2, PAH1* knockdown and further genetic modifications. Solely overexpressing the two plant proteins increased ABA titers by more than fivefold.

**Conclusions:**

In this report we were able to improve ABA titers and furthermore provide valuable insights for engineering other cell factories containing cytochrome P450 monooxygenases. Our results demonstrate that expressing heterologous plant proteins can be a simple yet highly effective engineering strategy to increase P450 monooxygenase activity.

**Supplementary Information:**

The online version contains supplementary material available at 10.1186/s12934-025-02913-8.

## Background

The isoprenoid abscisic acid (ABA) has become a molecule-of-interest for a multitude of applications in agriculture and medicine as well as nutrition. Its central role in plant physiology has long been known and its complex physiological interactions are still being investigated [1, 2]. Agricultural applications of ABA range from alleviating various abiotic stresses to regulating seed dormancy, germination and fruit ripening [3]. ABA can also act as a signalling molecule in organisms other than plants [4]. In recent years, ABA was shown to have various biological activities in mammals, making it a promising drug candidate. For example, pharmacological activity against various inflammatory diseases, pathogen-mediated infections, type-2-diabetes and metabolic syndrome has been reported [5–7]. ABA was furthermore identified as a bitter receptor blocker with potential applications in nutritional products [8].

**Fig. 1:**
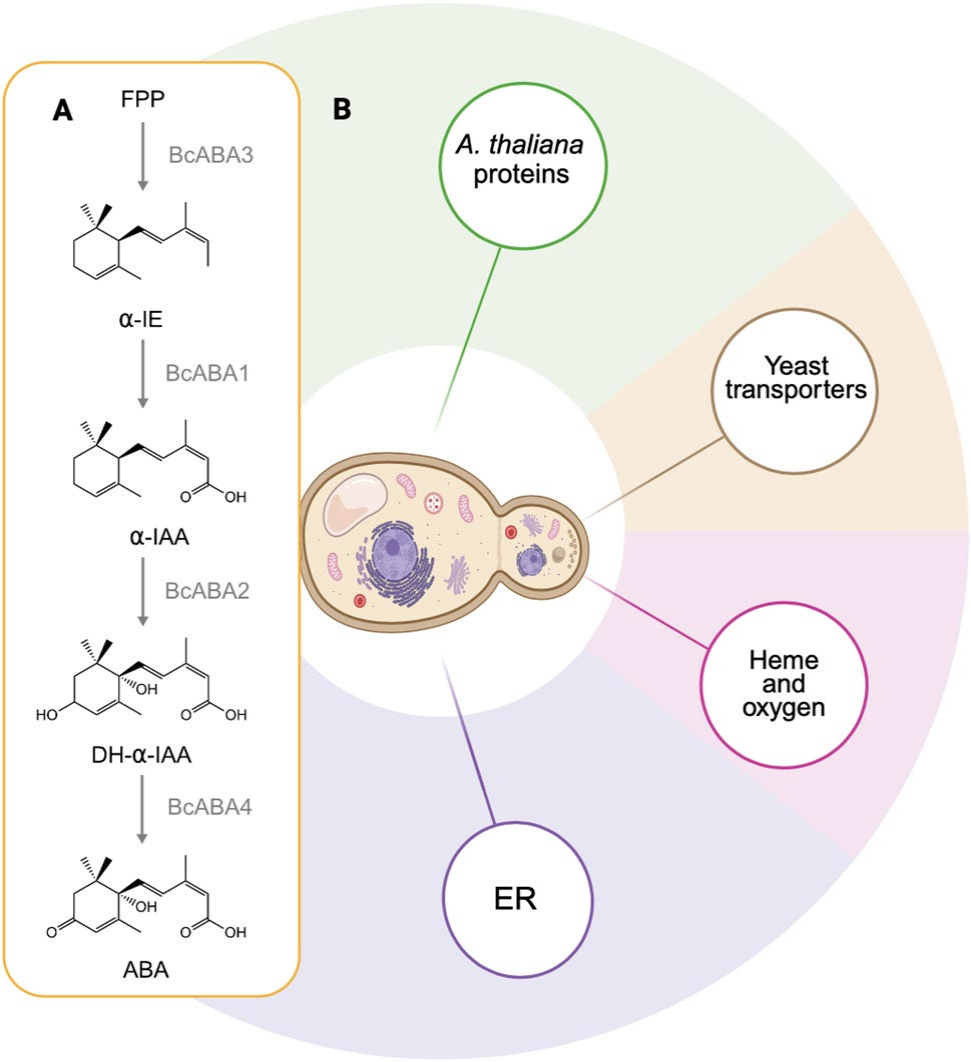
Overview of the engineered ABA production in *S. cerevisiae*. **A** ABA pathway in *Botrytis cinerea* [14]. The four pathway genes *bcaba1*, *bcaba2*,* bcaba3* and *bcaba4*, as well as the *B. cinerea* cytochrome P450 reductase encoding gene *bccpr1* were expressed in yeast to enable heterologous ABA production from the native precursor FPP [15]. **B** Schematic representation of different strategies explored in this study to enhance ABA production. Image created with BioRender.com. *FPP* farnesyl pyrophosphate, *α-IE* α-ionylideneethane, *α-IAA* α-ionylideneacetic acid, *DH-α-IE* 1′,4ʹ-trans-dihydroxy-α-ionylideneacetic acid, *ABA* abscisic acid, *ER* endoplasmic reticulum

A sustainable and inexpensive product source will be required to utilise ABA in these applications. The plant pathogenic fungus *Botrytis cinerea* is a natural producer of ABA and has been used as a biotechnological production host [9]. In *B. cinerea*, ABA is produced via the mevalonate (MVA) pathway from farnesyl-pyrophosphate (FPP) [10]. FPP is cyclised by the enzyme BcABA3 and subsequently oxidised at multiple positions by BcABA1, BcABA2 and BcABA4 to form ABA (Fig. 1A) [11–14]. However, the shortage of genetic tools for *B. cinerea*, as well as its hyphal morphology and comparatively slow growth make the fungus a challenging cell factory.

*Saccharomyces cerevisiae* has long been a workhorse for biotechnological applications. Its capabilities for high-level production of sesquiterpenes or sesquiterpenoids have been demonstrated previously, e.g. for the biofuel farnesene [16] or the anti-malarial drug precursor artemisinic acid [17]. In a previous study, we established a *S. cerevisiae* ABA cell factory by introducing the ABA pathway enzymes *Bc*ABA1, *Bc*ABA2, *Bc*ABA3 and *Bc*ABA4 as well as the *B. cinerea* cytochrome P450 reductase *Bc*CPR1 [15]*.* We demonstrated that two cytochrome P450 monooxygenases (CYPs, putatively of class II), *Bc*ABA1 and *Bc*ABA2, are limiting ABA titers in the current strain [15]. This pathway bottleneck was also observed in following studies [18–20].

CYPs are a large superfamily of enzymes that catalyse oxidation reactions using molecular oxygen and contain heme as a co-factor [21]. They are ubiquitous in plant and microbial biosynthetic pathways and have become a major engineering target in biotechnology [22, 23]. Class II CYP enzymes, the most common class in eukaryotes, require cytochrome P450 reductases (CPRs) as co-enzymes for the transfer of electrons from NADPH [24]. It was also shown that co-expression of cytochrome b5 (CYB5) and the cognate reductase (CBR) can have a positive impact on CYP activities, most likely by acting as additional electron donors [17, 25, 26].

Eukaryotic CYPs, CPRs, CYB5s and CBRs are usually anchored in the ER membrane which poses additional engineering challenges. It is presumed that available membrane space and/or its lipid composition significantly affect enzyme abundance and activity in the cell [22, 27]. Therefore, modulation and expansion of the ER membrane, also referred to as ER proliferation, has become a common engineering strategy for membrane-associated enzymes like CYPs and their co-enzymes. Various native target genes, most commonly *PAH1*, *INO2* and *OPI1*, have been investigated regarding ER proliferation, some of which led to multi-fold titer increases [28, 29]. *PAH1* encodes phosphatidate phosphatase, a highly regulated enzyme catalysing the conversion of phosphatidates to diacylgylcerols, a key reaction for balancing levels of membrane phospholipids (PL) and triacylglyceride (TAG) storage lipids [30]. Ino2 and Opi1 are transcription factors regulating various lipid metabolism genes. The Ino2/Ino4 complex activates genes involved in PL biosynthesis, whereas Opi1 acts as a repressor when interacting with Ino2 [31]. Knockout of *PAH1* or *OPI1* and overexpression of *INO2* result in similar phenotypes with an enlarged ER [28, 29, 32]. However, the effect of individual target genes appears to be strain- or product-specific.

Other strategies to optimize CYP activity include improving heme supply (an essential co-factor of CYPs, CPRs and CYB5s) or improving the availability of dissolved oxygen [20, 33, 34]. Overexpression of coproporphyrinogen III oxidase gene *HEM13* and deletion of the heme oxygenase gene *HMX1* appear to be promising targets to increase heme production [35]. Heterologous expression of the bacterial hemoglobin gene from *Vitreoscilla stercoraria*, *vsvhb*, has been investigated before with the goal of enhancing oxygen uptake and utilization in cell factories [36, 37]. *Vsvhb* expression furthermore benefited cell growth and protein synthesis in a variety of hosts [38].

Jiang and co-workers [39] adopted yet another approach to improve the activity of heterologous CYPs. By screening a cDNA library of *Arabidopsis thaliana,* they identified genes that positively affected two different CYPs, one involved in betaxanthin production, and one involved in α-santalol production. Specifically, the membrane steroid binding protein 1 (*At*MSBP1), the CONSTANS-like protein 4 (*AtCOL4*) and the glycine-rich RNA-binding protein 7 (*At*GRP7) proved to be beneficial. *At*MSBP1 is localized in the ER membrane and is suggested to have a chaperone-like function [40, 41]. *At*MSBP1 also physically interacts with three monolignol biosynthetic CYPs, forming a complex on the ER membrane also known as a metabolon, which regulates the lignin biosynthetic process [40]. This organization presumably enhances enzyme stability and activity, for example through substrate channelling. *AtCOL4* encodes a nuclear-localized protein that acts as a transcriptional repressor of flowering genes [42]. *AtGRP7* encodes a glycine-rich RNA-binding protein that can facilitate alternative splicing for various mRNAs. It is involved in physiological processes such as promoting flowering, enhancing the innate immune system and stress response [43]. How *At*COL4 *and At*GRP7 influence CYP activity is so far not elucidated.

Another engineering approach that is not directly related to CYPs but that was proven worthwhile in other cell factories that produce acidic compounds is modulating transporter expression. When produced in yeast, ABA is predominantly found in the cell culture supernatant [15]. As a weak organic acid with a *pK*_a_ of 4.75 [44], ABA is mostly present as the conjugate base in the cytosol of plants, with the membrane permeability of the ion being much lower than the protonated form [45, 46]. In yeast, ABA is presumably exported by an unspecific transporter. Complex regulatory networks mediate resistance to weak organic acids in *S. cerevisiae* with a multitude of transporters being involved in this process [47]. Upregulation of native transporters has been investigated for other cell factories with the goal of mediating product-related cell stress, such as increased turgor pressure or oxidative stress [47]. Nonetheless, downregulation of transporters could also be a valid engineering target by preventing the export of pathway intermediates, thereby increasing their local concentration in the cytosol. Identifying transporters involved in the import or export of a given heterologous compound is challenging. An alternative approach is to overexpress or knock-out global regulators involved in transporter expression. The transcription factors Pleiotropic Drug Resistance 1 (Pdr1) and Yeast Reveromycin-A Resistant 1 (Yrr1) are two such global regulators, involved in the pleiotropic drug resistance signalling network [48, 49]. Modulation of the PDR regulatory network could provide valuable insight for ABA-producing yeast strains and other *S. cerevisiae* cell factories producing weak organic acids.

Our previous proof-of-concept study demonstrated the feasibility of an *S. cerevisiae* cell factory, but ABA titres remained low. In this study, we aimed to overcome this limitation through rational engineering. We explore a variety of approaches including the modification of native gene targets and expression of heterologous proteins (Fig. 1B).

## Methods

### Plasmid construction

For PCR reactions, PrimeSTAR HS DNA Polymerase (Clontech), Phusion High-Fidelity DNA polymerase (Thermo Fisher Scientific) or SapphireAmp (Takara Bio) were used, and the manufacturer’s instructions were followed. Primer sequences can be found in Supplementary Table S1 (Additional file 1) and specific conditions of standard PCR reactions are listed in Supplementary Table S2 (Additional file 1). For plasmid and PCR product purification, GeneJet Purification Kits (Thermo Fisher Scientific) were used. Sanger DNA sequencing was performed by Eurofins Genomics. Primers and other oligonucleotides were ordered from Eurofins Genomics or Integrated DNA Technologies.

Plasmids used in this study are listed in Table 1. For construction of pMG138 the backbone of pCfB2312 was amplified by PCR using primers F-HR fwd and R-HR rev. The gRNA expression cassettes targeting *YRR1* and *PDR1* were ordered as gene fragments. Subsequently, pMG138 was constructed by Gibson assembly using the PCR product and the gene fragment.

**Table 1:** Plasmids used in this study

Plasmid name	Use	Backbone	Expression cassette	Reference
p416TEF	Episomal expression	–	Empty	[50]
p413TEF	Episomal expression	–	Empty	[50]
pRS316 + prTEF1 + HEM13 + terADH1	Episomal expression	pRS316	p*TEF1*-*HEM13-tADH1*	[35]
pPM28	Episomal expression	pRS316	pTDH3-eroGFP*-tCYC1*	[51]
pSPGM-INO2	Episomal expression	pSP-GM1	p*TEF1*-*INO2*-t*ADH1*	[52]
p423-INO2(L119A)	Episomal expression	p423 + TDH3	p*TDH3*-*INO2*(L119A)-*tCYC1*	This study
p426-ICE2	Episomal expression	p426 + TDH3	pTDH3-ICE2*-tCYC1*	This study
pCfB2904-ABA1-CPR	Genomic integration in XI-3	pCfB2904	p*PGK1*-*bcaba1*-t*ADH1* p*TEF1*-*bccpr1*-t*CYC1*	[15]
pCfB2909-ABA2-ABA4	Genomic integration in XII-5	pCfB2909	p*PGK1-bcaba2-*t*ADH1* p*TEF1*-*bcaba4-*t*CYC1*	[15]
pCfB3035-ABA3	Genomic integration in X-4	pCfB3035	p*TEF1*-*bcaba3*-t*CYC1*	[15]
pX3-bcaba1 + 2	Genomic integration in X-3	pMC-X3	p*TDH3*-*bcaba1*-t*TDH1* p*CCW12*-*bcaba2*-t*ENO2*	This study
pXII2-bccyb5 + cbr1	Genomic integration in XII-2	pMC-XII2	p*HHF2*-*bccyb5*-t*PGK1* p*TEF2*-*bccbr1*-t*SSA1*	This study
pX2-atcol4	Genomic integration in X-2	pMC-X2	p*HHF2*-*AtCOL4-tPGK1*	This study
pX2-msbp1	Genomic integration in X-2	pMC-X2	p*TEF2*-*AtMSBP1-tENO2*	This study
pX2-atcol4/atmsbp1	Genomic integration in X-2	pMC-X2	p*HHF2*-*AtCOL4-tPGK1* p*TEF2-AtMSBP1-tENO2*	This study
pXI2-vsvhb	Genomic integration in XI-2	pMC-XI2	p*CCW12*-*vsvhb-tTDH1*	This study
pXI5-ERG20	Genomic integration in XI-5	pMC-XI5	*pTEF2-ERG20-tTDH1*	This study
pCfB2312	Cas9 expression		*pTEF1-Cas9-tCYC*	[53]
pCfB3020	gRNA expression for X-2 integration			[53]
pCfB3041	gRNA expression for X-3 integration			[53]
pCfB3044	gRNA expression for XI-2 integration			[53]
pCfB3046	gRNA expression for X-5 integration			[53]
pCfB3051	gRNA expression for X-3, XI-2, XII-2 integration			[53]
pMG138	gRNAs expression targeting *PDR1* and *YRR1*	pCfB2312	p*TEF1*-*Cas9*-t*CYC1* p*SNR52-gRNA-tSUP4*	This study
pWS172/pWS158	Cas9 expression and gRNA expression targeting *HIS3*, *IRE1*, *INO2*, *HMX1*, *HAC1*	pWS158 (Addgene ID: 90517) pWS172 (Addgene ID: 90519)	*pPGK1-Cas9-tPGK1* *ptRNAPhe-gRNA-tSNR52*	This study
pMEL10	gRNA expression backbone used for knockout of *PAH1* and *OPI1*		p*SNR52-gRNA-tCYC1*	[54]

The Golden Gate-based MoClo workflow [55–57] was followed to construct pX3-bcaba1 + 2, pXII2-bccyb5 + cbr1, pX2-atcol4, pX2-atmsbp1, pX2-atcol4 + atmsbp1, pXI2-vsvhb and pXI5-ERG20. All MoClo assemblies are listed in Supplementary Table S3 (Additional file 1). First, primers pairs 306/357 and 307/310 were used to remove a BsaI site from *bcaba1* and to attach MoClo type-3 compatible overhangs via PCR. The resulting PCR products were fused together via a 2-step PCR reaction. In the first reaction, the PCR products were mixed in a 1:1 ratio (≈100 ng) with PrimeStar PCR master mix but without primers (55 °C T_ann_, 1:15 min t_elo_, 15 cycles). In the second reaction, 2 µL of the first reaction was used as template with the primer pair 306/307 (55 °C T_ann_, 1:40 min t_elo_, 35 cycles). The PCR product of the second reaction was purified and sequence verified. Primer pairs 308/309 were used to attach type-3 MoClo overhangs to *bcaba2*. DNA sequences lacking BsaI, BsmbI and NotI sites for *bccyb5* (UniProt identifier A0A384K1M2), *bccbr1* (UniProt identifier A0A384JNH0), *AtCOL4* (Uniprot identifier Q940T9), *AtMSBP1* (UniProt identifier Q9XFM6) and *vsvhb* (UniProt identifier P04252) were codon-optimised for yeast and provided by Genscript Biotech Corp. Primer pairs 292/293 and 294/295 were used to attach MoClo type-3 overhangs to *bccyb5* and *bccbr1*, respectively.

The MoClo compatible *bcaba1*, *bcaba2, bccyb5* and *bccbr1* fragments were inserted into the MoClo entry vector pYTK001 [56] (Supp. Table S3; Additional file 1). For *AtCOL4*, *AtMSBP1* and *vsvhb*, the strong Kozak sequence (TATACA) and type-3b overhangs were already included in the respectively cloning vector (pUC57-BsaI and BsmBI-free) containing each gene. Primer pairs oSMC 35/36, 37/38 and 39/40 were used to attach MoClo type-3a overhangs to the *HHF2*, *TEF2* and *CCW12* promoters, respectively.

Subsequently, level-1 MoClo plasmids were assembled for each gene. The level-1 plasmids were combined with the backbones pMC-X3, pMC-XII2. pMC-X2 and pMC-XI2 [57] to form pX3-bcaba1 + 2, pXII2-bccyb5 + cbr1, pX2-atcol4, pX2-atmsbp1, pX2-atcol4 + atmsbp1 and pXI2-vsvhb respectively.

For the construction of pXI5-ERG20, genomic DNA from CEN.PK113-5D was used as a template. Primer pairs oSMC123/124 were employed to attach type-3 MoClo overhangs to *ERG20*. The purified DNA fragment was then combined with pYTK014, pYTK056, and the preassembled level-2 MoClo backbone, pMC-XI5 [57], to create pXI5-ERG20 (Supp. Table S3; Additional file 1). To generate p426GPD-INO2 (L119A) and p426-ICE2, *INO2 *(L119A) was amplified from genomic DNA using the primer pair oSMC 25/26 with the template being the PCR product obtained from the colony that was successfully mutated and prior to the plasmid curation process, as is explained in the results. The *ICE2* coding sequence was amplified from genomic DNA of CEN.PK113-11C using the primer pair oSMC 27/28. Both obtained PCR products were digested with the restriction enzymes XmaI and XhoI before being ligated into the corresponding restriction sites in p423GPD and p426GPD plasmids [50]. Primer pair oSMC 78/84 was used for sequencing and validation of the cloning of p423-INO2 (L119A) and p426-ICE2.

### Microorganisms and media

Plasmids were used to transform NEB® 5-alpha competent *E. coli* cells (New England Biolabs). *E. coli* was cultivated at 37 °C in liquid lysogeny broth (LB) medium or on LB agar plates with appropriate antibiotics. *S. cerevisiae* strains are listed in Table 2 and a pedigree chart is shown in Supplementary Figure S1 (Additional file 1). *E. coli* was cultivated at 37 °C in liquid lysogeny broth (LB) medium or on LB agar plates with appropriate antibiotics. Yeast was cultivated at 30 °C in liquid yeast extract peptone dextrose (YPD) medium or mineral medium (adapted from [58]) while shaking at 220 rpm. For cultivation on agar plates YPD medium or synthetic defined (SD) medium was used. For all yeast media, 20 g/L glucose was used. Unless stated otherwise, SD medium was supplemented with 100 mg/L uracil or histidine for strains with uracil or histidine auxotrophy. Detailed media compositions can be found in Supplementary Table S4 (Additional file 1).

**Table 2:** Strains used and constructed in this study

Strain name	Parent strain	Genomic modifications	Plasmid	Reference
CEN.PK113-5D	–	*MAT*a *MAL2*-*8* ^*c*^ *SUC2 ura3*-*52*	–	[59], provided by P. Kötter, University of Frankfurt, Germany
CEN.PK113-11C	–	*MAT*a *MAL2*-*8* ^*c*^ *SUC2 ura3*-*52 his3Δ*	–	provided by P. Kötter, University of Frankfurt, Germany
yMG01	CEN.PK113-11C	p*TEF1*-*YRR1* p*TDH3*-*PDR1*	–	This study
yMG02	CEN.PK113-11C	*yrr1*Δ *pdr1*Δ	–	This study
SCIGS22a	CEN.PK113-5D	*lpp1*Δ::*loxP dpp1*Δ::*loxP* p*ERG9* Δ::*loxP* p*HXT1* *gdh1*Δ::*loxP* p*TEF1*-*ERG20* p*PGK1*-*GDH2* p*TEF1*-*tHMG1*	–	[60]
SABA3	SCIGS22a	p*PGK1*-*bcaba1* p*PGK1-bcaba2* p*TEF1*-*bcaba3* p*TEF1*-*bcaba4* p*TEF1*-*bccpr1*	–	[15]
yMO22	SABA3	p*TDH3*-*bcaba1* p*CCW12*-*bcaba2*	–	This study
yMO23	yMO22	p*HHF2*-*bccyb5* p*TEF2*-*bccbr1*	–	This study
yMO26	yMO23	*pah1*Δ	–	This study
yMO35	yMO26	–	p416TEF	This study
yMO36	yMO23	p*PAH1*Δ::p*REV1*	–	This study
yMO38	yMO23	p*PAH1*Δ::p*HXT1*	–	This study
yMO39	yMO23	p*PAH1*Δ::p*minCYC1*	–	This study
yMO40	yMO23	–	pRS316 + prTEF1 + HEM13 + terADH1	This study
yMO41	yMO23	–	pSPGM-INO2	This study
yMO42	yMO23	–	p416TEF	This study
yMO48	SABA3	–	p416TEF	This study
yMO49	yMG01	p*PGK1-bcaba1* p*PGK1-bcaba2* p*TEF1-bcaba3* p*TEF1-bcaba4* p*TEF1-bccpr1*	p416TEF, p413TEF	This study
yMO50	yMG02	p*PGK1-bcaba1* p*PGK1-bcaba2* p*TEF1-bcaba3* p*TEF1-bcaba4* p*TEF1-bccpr1*	p416TEF, p413TEF	This study
yMO51	yMO23	*opi1*Δ	p416TEF	This study
ySMC001	SABA3	*his3*Δ	–	This study
ySMC003	CEN.PK113-11C	*INO2 (L119A)*	–	This study
ySMC017	ySMC001	*ino2*Δ	p423TDH3-INO2 (L119A) p426TDH3-ICE2	This study
ySMC019	ySMC001	*ire1*Δ *HAC1i*	pPM28	This study
ySMC020	ySMC001	*hmx1*Δ	pRS316 + prTEF1 + HEM13 + terADH1	This study
ySMC023	ySMC001	p*TEF2*-*AtMSBP1*	–	This study
ySMC024	ySMC001	p*HHF2*-*AtCOL4*	–	This study
ySMC025	ySMC001	p*HHF2*-*AtCOL4* p*TEF2*-* AtMSBP1*	–	This study
ySMC026	ySMC025	*pTEF2*-*ERG20*	–	This study
ySMC027	yMO39	p*TEF2*-*AtMSBP1*	–	This study
ySMC028	yMO39	p*HHF2*-*AtCOL4* p*TEF2*-* AtMSBP1*	–	This study
ySMC029	ySMC028	*pTEF2*-*ERG20*	–	This study
ySMC030	ySMC001	p*CCW12*-*vsvhb*	–	This study

### Strain construction

Yeast strains were transformed according to the protocol by Gietz and Woods [61]. The plasmids pX3-bcaba1 + 2, pXII2-bccyb5 + cbr1, pX2-atcol4, pX2-atmsbp1, pX2-atcol4 + atmsbp1, pXI2-vsvhb and pXI5-ERG20 were used for genomic integrations. The procedure described in Jessop‐Fabre et al. [53] was followed, using the Cas9-encoding plasmid pCfB2312 and the gRNA-helper plasmids pCfB3020, pCfB3041, pCfB3044, pCfB3046 and pCfB3051.

For the *PDR1* and *YRR1* deletion or overexpression, gRNAs were designed using Benchling. These gRNAs targeted sites upstream of the start ATG of the respective ORF, so that the same gRNA could serve for both deletion and overexpression purposes. The repair fragments for deletion of *YRR1* and *PDR1* were ordered as 120-bp oligonucleotides (Supp. Table S5, Additional File 1). Hybridized fragments were used along with pMG138 to transform CEN.PK113-11C. For promoter replacement, the promoter sequences p*TDH3* and p*TEF1* were amplified from genomic DNA of CEN.PK113-11C using primers pTDH3-PDR1 fwd + rev and pTEF1-YRR1 fwd + rev, respectively. Homology to *PDR1* and *YRR1* was introduced by reamplifying p*TDH3* and p*TEF1* with primers pTDH3-PDR1 ampl OL fwd + rev and pTEF1-YRR1 ampl OL fwd + rev, respectively. Purified fragments were used along with pMG138 to transform CEN.PK113-11C. Obtained clones were verified by colony PCR using primers PDR1 fwd + rev and YRR1 fwd + rev, respectively.

*PAH1* and *OPI1* were deleted following the protocol by Mans et al. [54], using the plasmid pMEL10 for gRNA expression and the Yeastriction tool (http://yeastriction.tnw.tudelft.nl/). The sequences of the gRNA fragments and repair oligos can be found in Supplementary Table S5 (Additional file 1). Primers pairs 366/367 and 1260/1261 (Supp. Table S1 and Supp. Table S2; Additional file 1) were used to validate the *pah1*Δ and *opi1*Δ genotype, respectively.

For the *PAH1* promoter replacement, the gRNA was designed using the Benchling CRISPR Guide tool (Supp. Table S5) and its coding sequence inserted into pMEL10. For construction of the linear promoter replacement cassettes, primer pairs 417/418, 422/423, 424/425 were used. The primers were designed to contain homologous regions for replacing 771 base pairs upstream of the *PAH1* start codon. The resulting PCR products were used as repair fragments in a transformation according to Mans et al. [54]. Primers 426/427 were used to validate the p*PAH1* replacement. The colony PCR product of strain yMO36 was sequenced for verification.

To delete *HIS3*, *IRE1*, *INO2* and *HMX1*, the protocol described by Shaw et al. [62] was used. Briefly, two oligonucleotides encoding the gRNA were annealed in vitro and integrated into the CRISPR/Cas9 expression plasmid pWS158 or pWS172 using a BsmBI Golden Gate assembly. Primer pairs oSMC: 57/58, 29/30, 1/2 and 48/49, were used to generate sgRNA for *HIS3*, *IRE1*, *INO2* and *HMX1,* respectively.

The constructed plasmids pWS158 or pWS172were used to transform the respective yeast strain together with the donor DNA to facilitate homology-directed repair at the double-strand break. Donor DNA was created by no-template PCR of partially overlapping primers, consisting of the 60 nucleotides upstream of the start codon, followed by the 60 nucleotides downstream of the stop codon, as previously described [63]. The DNA sequences can be found in Supplementary Table S5 (Additional file 1). Primer pairs oSMC: 89/90, 11/14, 5/6 and 59/60 were used were used to validate the *his3*Δ, *ire1*Δ, *ino2*Δ and *hmx1*Δ genotypes, respectively.

To introduce the point mutation L119A in *INO2*, CEN.PK113-11C was transformed with pWS158 expressing a gRNA targeting the locus and the donor DNA containing the desired mutation (together with a synonymous mutation in the target sequence to prevent Cas9 cleavage after repair) [63]. Primer pairs oSMC 1/2 and oSMC 5/6 were used to generate gRNA and for sequence validation, respectively.

To obtain the *HAC1i* genotype, the gRNA-Cas9 expression plasmid pWS172 was cotransformed with the donor DNA. The gRNA targeting the *HAC1* intron was generated by annealing the oligos oSMC33 and oSMC34. The donor DNA for repair consisted of the spliced *HAC1* version (*HAC1i*), obtained by fusing two DNA fragments of *HAC1* using primer pairs oSMC 15/16 and oSMC 17/18 through overlap extension PCR. Primers oSM31 and oSM32 were used to validate the intron deletion by colony PCR and sequencing.

Codon-optimized sequences of heterologous genes used in this study can be found in Supplementary Table S6 (Additional file 1).

### Cultivation for ABA analysis

Single colonies were picked from agar plates for precultures (1.5 mL mineral media) in 14 mL round-bottom cultivation tube (Greiner Bio-One). Precultures were grown for 48 or 72 h (for slow growing *pah1*Δ strains) and main cultures (2.5 mL mineral media) were inoculated at OD_600_ 0.1 in 24-deepwell microplates (square wells, pyramid-bottom, EnzyScreen). Cultures were grown for 48 h (Figs. 3, 4) or 60 h (Fig. 5), OD_600_ was measured, cultures were centrifuged (5 min, 1500×*g*) and 1 mL supernatant was transferred to 2-mL Eppendorf tubes.

### ABA extraction and quantification

**Fig. 2:**
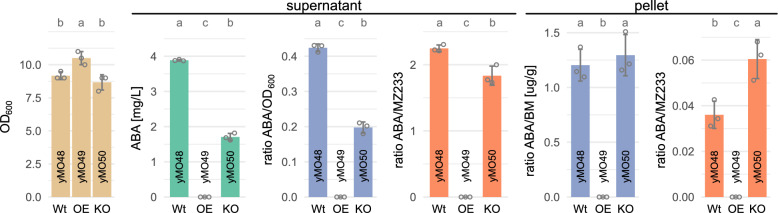
Effects of native transporter expression modulation in ABA-producing strains. Genes encoding the transcription factors Pdr1 and Yrr1 were either overexpressed (yMO49) or knocked out (yMO50) and compared to a control strain with wild-type *PDR1* and *YRR1* expression (yMO48). OD_600_ (*beige*), ABA titer (*green*), ABA titer normalized to OD_600_ or biomass (*blue*), and ion count ratio of ABA to unknown metabolite MZ233 (*orange*) are shown after 48 h of cultivation in mineral medium (24-deepwell microplates). Supernatant and cell pellet were analysed separately. Grey circles show the individual data points used to calculate the mean and standard deviation. Only traces of ABA (<0.05 mg/L) and MZ233 were detected for yMO49. Significance is shown as compact letter display above the bars. Shared letters indicate no significant difference. *Wt* wild-type, *OE* overexpression, *KO* knockout

**Fig. 3:**
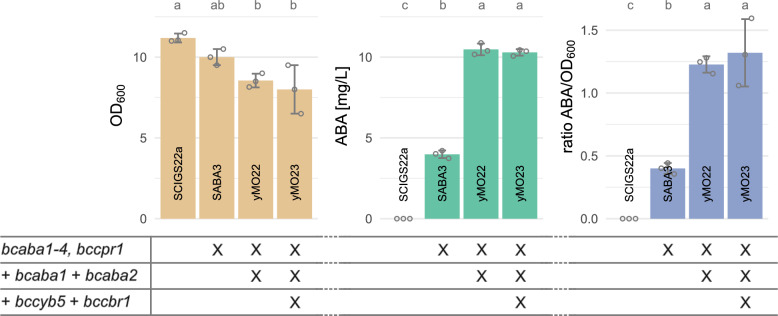
Effects of expressing additional copies of *bcaba1* and *bcaba2*, as well as expressing the *B. cinerea* CYB5 (*bccyb5*) and its cognate CBR (*bccbr1*). “X” indicates presence of the genetic modification in the strain. OD_600_ (*beige*), ABA titer in the supernatant (*green*) and ABA titer normalized to OD_600_ (*blue*) are shown after 48 h of cultivation in mineral medium (24-deepwell microplates). Grey circles show the individual data points used to calculate the mean and standard deviation. Strain SCIGS22a not containing any ABA pathway genes was used as control. Significance is shown as compact letter display above the bars. Shared letters indicate no significant difference

**Fig. 4:**
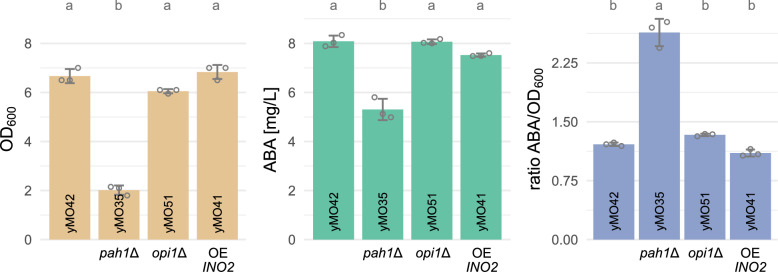
Effects of *pah1*Δ, *opi1*Δ or *INO2* overexpression on ABA production. Genetic modifications additional to yMO42 (yMO23 carrying an empty plasmid) are displayed below the bars and include knockout of *PAH1* (yMO35) or *OPI1* (yMO51) and episomal overexpression of *INO2* (yMO41). OD_600_ (*beige*), ABA titer in the supernatant (*green*) and ABA titer normalized to OD_600_ (*blue*) are shown after 60 h of cultivation in mineral medium (24-deepwell microplates). Grey circles show the individual data points used to calculate the mean and standard deviation. Significance is shown as compact letter display above the bars. Shared letters indicate no significant difference. *OE* overexpression

**Fig. 5:**
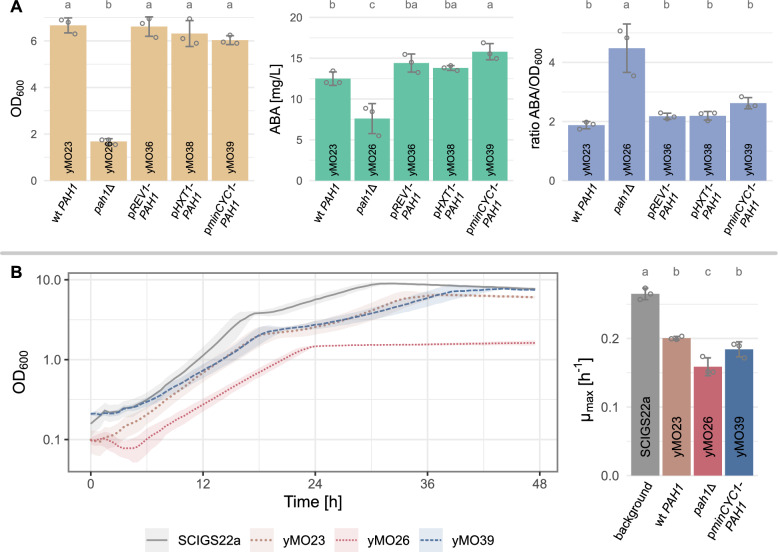
Effects of *PAH1* promoter replacement. The *PAH1* promoter was exchanged for p*REV1* (yMO36), p*HXT1* (yMO38) or p*minCYC1* (yMO39) and the effect was compared to the parent strain with wild-type p*PAH1* (yMO23) or a *pah1*Δ strain (yMO26). Grey circles show the individual data points used to calculate the mean and standard deviation. Significance is shown as compact letter display above the bars. Shared letters indicate no significant difference. *Wt* wild-type. **A** OD_600_ (*beige*), ABA titer in the supernatant (*green*) and ABA titer normalized to OD_600_ (*blue*) are shown after 60 h of cultivation in mineral medium (24-deepwell microplates). **B** Growth profiles and maximum growth rate μ_max_ are shown for the background strain SCIGS22a (no *B. cinerea* genes, *grey*), the wild-type *PAH1* control yMO23 (*brown*), the *pah1*Δ strain yMO26 (*red*) and yMO39 carrying the p*minCYC1*-*PAH1* modification (*dark blue*). Lines in the growth profiles show the mean OD_600_ of three replicates and ribbons visualize their standard deviation. Cells were grown in mineral media (96-well microplates)

For Figs. 2, 3, 4 and 5, ABA was extracted similarly to the procedure described in [15]. One mL of ethyl acetate (>99.9% Sigma-Aldrich) containing 0.5% (v/v) formic acid (>98%, Sigma-Aldrich) was added to the 2-mL Eppendorf tubes containing 1 mL culture supernatant. The tubes were vortexed for 10 s, then centrifuged (10 min, 13,000×*g*, 4 °C), and 0.8 mL of the supernatant was transferred to a new Eppendorf tube. The solvent was evaporated using a Genevac miVac (45 min, 20 mBar, 45 °C). The pellet was reconstituted in 0.8 mL methanol (>99.9%, Sigma-Aldrich), centrifuged (10 min, 13,000×*g*, 4 °C) and ≈0.5 mL of the supernatant was transferred to an HPLC (high-performance liquid chromatography) vial.

**Fig. 6:**
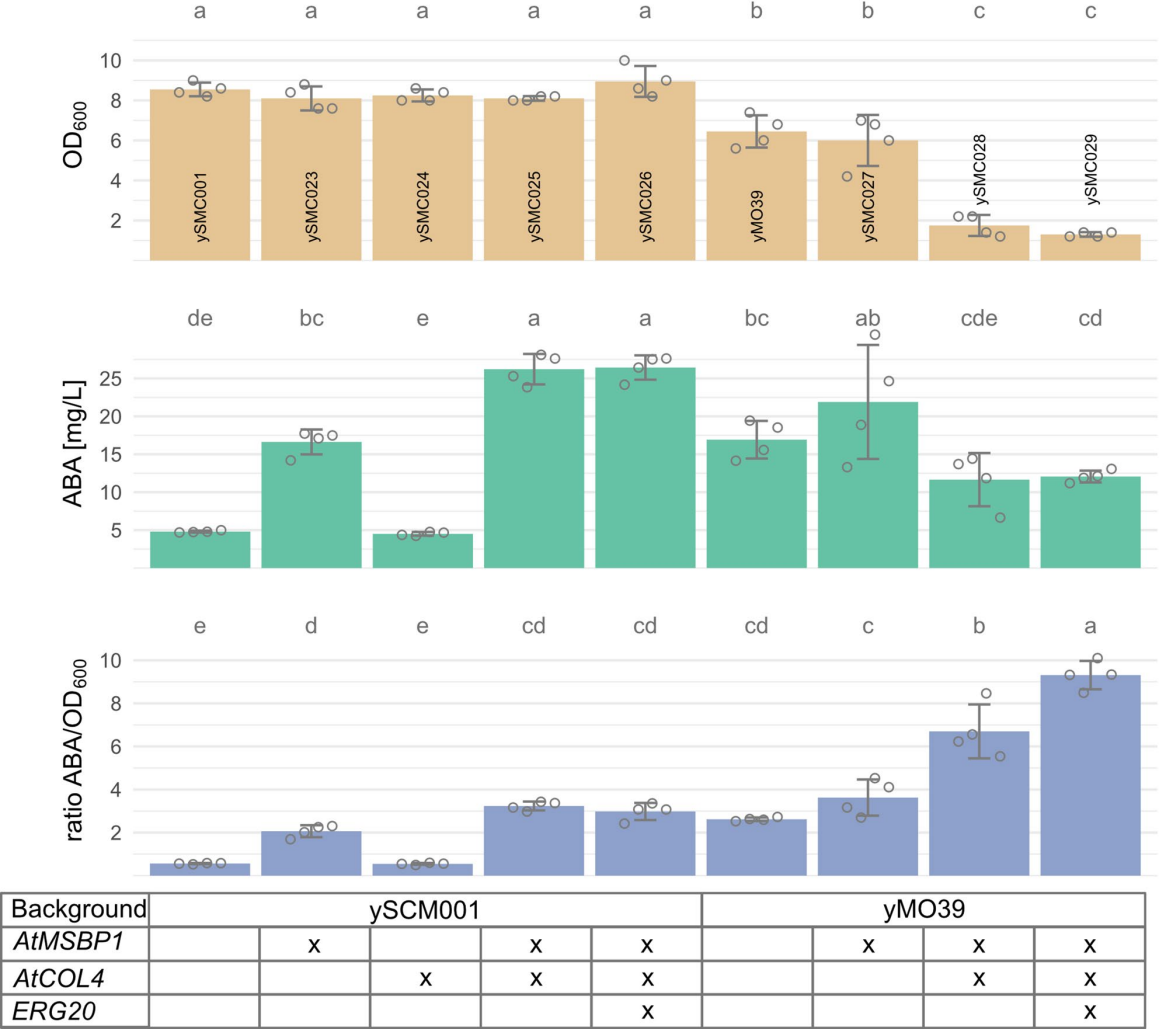
Effects of *AtMSBP1* and *AtCOL4* expression and *ERG20* overexpression. The *Arabidopsis* genes were integrated in the ySCM001 background (single copies of *bcaba1/2/3/4* and *bccpr1*) or yMO39 background (two copies of *bcaba1/2*, expression of *bccyb5* and *bccbr1* as well as the p*minCYC1*-*PAH1* modification). An additional copy of *ERG20* was added to improve precursor supply. “X” indicates presence of the genetic modification in the strain. OD_600_ (*beige*), ABA titer in the supernatant (*green*) and ABA titer normalized to OD_600_ (*blue*) are shown after 48 h of cultivation in mineral media (24-deepwell microplates). Grey circles show the individual data points used to calculate the mean and standard deviation. Significance is shown as compact letter display above the bars. Shared letters indicate no significant difference

A simpler sample preparation procedure was used for the experiments displayed in Fig. 6 and Supplementary Figures S4 (B, C) and S5 (Additional file 1). Cell cultures were centrifuged (5 min, 2000×*g*), then 1 mL of supernatant was filtered using a 0.22‐µm nylon filter and transferred to an HPLC vial.

The samples were analysed in an Agilent 6120 Single Quadrupole mass spectrometer (MS) with an Agilent Infinity 1260 HPLC system consisting of a binary pump, autosampler and thermostat column compartment. Ionization was performed using an atmospheric pressure electrospray ionization (API-ES) source (positive mode). Compounds were separated on an Agilent Poroshell 120 EC-C18 (2.7 μm, 3.0 × 50 mm) column (maintained at 40 °C) using a water-acetonitrile gradient with 0.04% formic acid in both solvents. The gradient started with 95% water and, over 5 min, gradually changed to 95% acetonitrile (> 99.5%, Sigma-Aldrich). After a 2-min hold, the gradient was ramped back to 95% water over 3 min. The sample injection volume was set to 10 µL. ABA was quantified by selected ion monitoring (m/z = 265) and (S)-(+)-ABA standard (>98%, Cayman Chemicals) was used to fit a calibration curve (2nd polynomial).

### Growth Profiler analysis

Growth (Fig. 5B) was analysed using a Growth Profiler 960 (Enzyscreen). Precultures were prepared as described above and cultures were subsequently grown in transparent bottom 96-well plate (Enzyscreen) with 250 µL mineral media per well. Pictures were taken every 30 min.

### Software and statistical analysis

Besides the manufacturer’s software for the Growth Profiler (Enzyscreen) and the HPLC–MS (Agilent), R Studio [64] was used to analyse the data. Relevant R packages include tidyverse [65], emmeans [66], multcomp [67] and growth rates [68]. Benchling (www.benchling.com) was used for planning and analysing DNA constructs. For supplementary figures S4 and S5 (Additional file 1) data were plotted using GraphPad Prism 9.5.1 (GraphPad Software).

One-way ANOVA followed by Tukey's honest significance test (⍺ = 0.05) was used to determine significant differences between the strains. Results are displayed as compact letter display above the bar charts. For two groups’ comparison of data, an unpaired student’s t-test was used.

## Results and discussion

### Effects of transporter expression modulation on ABA production

Overexpression or knock-down of native transporters could be beneficial for ABA production, either by alleviating product-related cell stress, as was shown for the anti-malaria drug precursor artemisinic acid [69], or by preventing the export of pathway intermediates.

To test the two hypotheses, the endogenous genes encoding two global transcriptional regulators involved in transporter expression, Yrr1 and Pdr1, were either overexpressed using strong, constitutive promoters, or deleted. Altered transporter activity in the resulting strains was confirmed by evaluating the response to the antifungal fluconazole. As expected, while overexpression of *PDR1* and *YRR1* resulted in increased fluconazole resistance, their deletion resulted in increased fluconazole sensitivity (Supp. Fig. S2, Additional file 1).

Next, the *B. cinerea* genes *bcaba1*, *bcaba2*, *bcaba3*, *bcaba4* and *bccpr1* were integrated in the strains’ genomes to establish ABA production. Figure 2 shows the measured OD_600_, ABA titer, ratio of ABA to OD_600_, and ratio of ABA to an unknown compound with the mass-to-charge (m/z) value 233.15, referred to as MZ233. MZ233 was earlier detected in the supernatant of ABA-producing strains and is assumed to be an intermediate or side-product in the ABA pathway [15]. ABA and MZ233 were quantified separately in the supernatant and cell pellet.

Small, but significant differences were detected in the OD_600_ at 48 h, with yMO49, overexpressing *PDR1* and *YRR1*, exhibiting a higher OD_600_ compared to the control, yMO48, and the double-knock-out strain, yMO50 (Fig. 2). Surprisingly, only traces of ABA and MZ233 were detected in the supernatant and pellet of the overexpression strain yMO49. About 50% less ABA was detected in the supernatant of the knock-out strain yMO50 compared to the control; however, the amount of ABA in the cell pellet remained the same. In the supernatant, the control strain showed a significantly higher ratio of ABA/MZ233 compared to yMO50, though the difference was only 20%. In contrast, the ABA/MZ233 ratio was 70% higher for yMO50 in the cell pellet. This shows that, when normalized to ABA, the double-knock-out strain contains more MZ233 in the supernatant and less MZ233 in the cell pellet.

Neither overexpression of *PDR1* and *YRR1*, nor their deletion improved ABA production. In the overexpression strain yMO49, ABA intermediates, likely upstream of MZ233, might be exported rapidly, thereby removing substrates from the product pathway and resulting in only traces being detectable. However, no additional peaks of postulated intermediates were identified in the HPLC–MS chromatogram of the supernatant when comparing yMO49 to yMO48 (Supp. Fig. S3, Additional file 1) and high-resolution MS analysis would be necessary to confirm this hypothesis. The increase in OD_600_ for yMO49 indicates that the strain experiences less weak-acid-related stress [47, 69]. The reasons for the reduced ABA titers in yMO50 compared to the wild-type control are unclear. Nonetheless, the higher intracellular ABA/MZ233 ratio for yMO50 (Fig. 2) suggests that, ratio wise, more MZ233 is converted to ABA in yMO50 (assuming that MZ233 is a pathway intermediate). The results indicate that one or multiple Pdr1/Yrr1-regulated transporters facilitate the transport of ABA pathway intermediates—if not of the product itself. Pdr1 and Yrr1 control the expression of several genes encoding ATP-binding cassette (ABC) transporters involved in the multidrug response in yeast, such as *YOR1*, *SNQ2*, *PDR5*, *PDR10* and *PDR15* (reviewed in [70]). Modulating individual transporters could still be worth investigating in future studies. This is exemplified by a study investigated the effect of expressing *A. thaliana* ABA transporters in an ABA-producing *Y. lipolytica* strain, with the aim of reducing cellular stress; however, ABA titers remained unchanged [18]. In another recent study native ABC transporters were overexpressed or deleted in an ABA-producing *Y. lipolytica* strain [71]. Specifically, overexpression of the gene encoding the transporter *Yl*Gcn20 (named due to its similarities to the *S. cerevisiae* protein Gcn20) improved ABA production by about 10%, while the overexpression of other transporters notably decreased ABA levels and/or biomass accumulation [71]. Wang et al. [72] demonstrated how disruption of the transportome in *S. cerevisiae* can be used to identify native genes involved in the transport of heterologous products. The same approach could be followed for an ABA-producing strain.

### Genomic integration of additional pathway gene copies and improving co-enzyme and co-factor availability

The previously engineered *S. cerevisiae* strain SABA3 was used as the starting strain in this study [15]. This strain is derived from the terpenoid platform strain SCIGS22a with increased flux through the MVA pathway and improved NADPH supply [60, 73], and contains single copies of the *B. cinerea* genes *bcaba1*, *bcaba2*, *bcaba3*, *bcaba4*, and *bccpr1* (Fig. 1A; Table 2). In our previous study, we showed that plasmid-based expression of additional gene copies increased the ABA titer more than fourfold [15]. Genomic integration of expression cassettes is mostly preferred to episomal expression for biotechnological applications, since it results in less cell-to-cell variability and higher genetic stability [56, 74]. Therefore, additional copies of *bcaba1* and *bcaba2* were integrated into the genome of SABA3 resulting in the strain yMO22. *Bcaba1* and *bcaba2* were expressed using the strong promoters p*TDH3* and p*CCW12*, respectively. Furthermore, we investigated the effect of expressing the *B. cinerea* cytochrome b5, encoded by *bccyb5*, and cognate cytochrome b5 reductase, encoded by *bccbr1*. The strain yMO23 (originating from yMO22) contains an expression cassette with p*HHF2*-*bccyb5* and p*TEF2*-*bccbr1* integrated in the genome.

As expected, the additional copies of *bcaba1* and *bcaba2* resulted in an increase in ABA production, both in the absolute titer and titer normalised to OD_600_ (Fig. 3). The 2.6-fold increase when comparing SABA3 and yMO22 was lower than the 4.1-fold increase that was previously observed in the plasmid-carrying strain [15]. This was anticipated since plasmid-based gene expression levels are often higher when compared to genomic integrations [56, 75], likely due to cells carrying on average more than one copy of centromeric plasmids [76]. These results indicate that CYP activity still limits ABA titers.

Conversely, expression of *bccyb5* and *bccbr1* in yMO23 did not result in an increase of ABA titers (Fig. 3) which highlights the complex roles of CYB5s. For some CYPs, the co-enzyme is required for the reaction to be catalysed while in other cases CYB5s have a modulating effect (stimulatory or inhibitory) [77].

We also tested if the supply of heme is limiting productivity; however overexpression of the coproporphyrinogen III oxidase gene *HEM13* did not improve ABA titers (Supp. Fig. S4A; Additional file 1) and only a slight improvement of 20% was observed when combined with the deletion of the heme oxygenase gene *HMX1* (Supp. Fig. S4B; Additional file 1). We also expressed *vsvhb* with the goal of improving oxygen availability. However, this did not benefit ABA titer nor cell growth (Supp. Figure S4 C; Additional file 1). These data indicate that oxygen availability and heme supply is sufficient in the current strain.

### Effects of ER proliferation on ABA production

ER proliferation is a promising engineering strategy for ABA cell factories since it was successfully applied for other isoprenoid-producing strains and does not require engineering of the heterologous CYP itself [28, 29]. Three of the most commonly targeted native genes for ER proliferation are *PAH1*, *INO2* and *OPI1*. We investigated the effects of a *PAH1* or *OPI1* knockout (strains yMO35 and yMO51 respectively) and *INO2* overexpression (strain yMO41). Even though the addition of *bccyb5* and *bccbr1* in yMO23 did not result in higher ABA titers (Fig. 3), we decided to use the strain for further experiments, since BcCYB5 activity could also be affected by ER expansion. *INO2* was overexpressed using a centromeric plasmid while the other strains analysed in this experiment were transformed with an empty plasmid to render them prototrophic.

Figure 4 shows the OD_600_, ABA titer and ABA titer normalized to OD_600_ for strains with engineered PL metabolism. OD_600_ and ABA titers were comparable for all strains, with exception of the *pah1*Δ strain yMO35. yMO35 exhibited a severe growth defect with ≈70% lower OD_600_ after 60 h compared to the control strain yMO42. Absolute ABA titers for yMO35 were decreased by ≈35%; however, the ABA titer relative to the OD_600_ was increased 2.2-fold. The results suggest that *pah1*Δ-mediated ER expansion was beneficial for ABA production. However, the positive effects of the *PAH1* knockout came with impaired growth, a phenotype that has been observed before [78–80].

We decided to also investigated other strategies to modulate the ER microenvironment including overexpression of *ICE2* (encoding a protein indirectly involved in Pah1 regulation), de-regulation of Ino2 via an L119A point mutation and provoking the ER-based unfolded protein response (UPR) (via simultaneous knockout of *IRE1*, *HAC1i* expression and eroGFP expression) in strain ySMC019 [81–84]. Interestingly, integrating the L119A mutation in *INO2* in the genome was highly unstable and reverted within 72 h (data not shown). Instead, we explored episomal expression of the L119A mutant. However, none of these modifications resulted in higher ABA titer (Supp. Fig. S5; Additional file 1). A higher ABA yield was observed for the strain with upregulated UPR, ySMC019, but the strain also exhibited a severe growth defect (Supp. Fig. S5; Additional file 1).

Even though modification of the target genes *PAH1*, *OPI1*, *INO2* and *ICE2* all resulted in ER expansion in other reports, they did not all affect CYP activity in the ABA-producing strains. A previous study compared the effects of *pah1*Δ, *opi1*Δ and overexpression of *INO2* for the production of isoflavonoids [85]. In that study, flavonoid titers were improved by either knocking out *OPI1* or by overexpressing *INO2*. No beneficial effects were observed for *pah1*Δ, and severe growth defects were only seen for a *pah1*/*opi1* double-knock-out strain and a strain with *opi1*Δ and simultaneous *INO2* overexpression [85]. Interestingly, another report showed that deletion of *PAH1* in *Y. lipolytica* resulted in an improvement in ABA production, whereas *INO2* overexpression or *OPI1* deletion had no effect [20]. This is in accordance with our observations in *S. cerevisiae* (Fig. 4).

As expected from previous reports, the beneficial effects of ER proliferation are strain- and/or product-specific, potentially depending on other genetic modifications or being specific to the heterologous CYP. In this context it is noteworthy that the ABA-producing strains of this study are based on the sesquiterpenoid platform strain SCIGS22a, in which the genes *DPP1* and *LPP1* were deleted to prevent the dephosphorylation of farnesyl-pyrophosphate to farnesol [60, 73, 86]. Like *PAH1*, *DPP1* and *LPP1* encode phosphatidate phosphatases. Dpp1 and Lpp1 only have minor effects on PL and TAG levels [87], but their deletion potentially influenced the growth and ABA titer of the *pah1*Δ strain yMO35.

### Exchanging of the native *PAH1* promoter to mediate growth deficiency

The deletion of *PAH1* in yMO35 led to a severe growth defect, making the strain unsuitable for further engineering or future biotechnological uses. However, encouraged by the improved relative ABA titer of yMO35 (Fig. 4), we investigated if a gene knock-down could mediate the growth defect and still benefit ABA production. Expression levels of the *PAH1* promoter during growth in glucose are comparable to the *REV1* promoter and are <1% of the commonly used strong *TEF1* promoter (unpublished data).

The native *PAH1* promoter was exchanged for three different promoters to investigate their effects on growth and ABA production. We chose the constitutively weak promoter p*REV1* (strain yMO36), the glucose-concentration-dependent promoter p*HXT1* (strain yMO38) and a synthetic minimal promoter named p*minCYC1* (strain yMO39) [88]. p*HXT1* is repressed in low glucose conditions and is used in the background strain SCIGS22a to regulate *ERG9* expression with the goal of separating cell growth and production phase [73, 89]. Different truncated versions of the *CYC1* promoter have been used in other studies before and minimal expression levels were observed [88, 90]. In our study, we used a substantially truncated version (144 bp, sequence in Supplementary Table S6, Additional file 1), in which only one of three TATA elements remain [88, 91].

Figure 5A shows the OD_600_, absolute and relative ABA titers of strains with replaced p*PAH1*, the control strain with wild-type p*PAH1* (yMO23) and the *pah1*Δ strain (yMO26). The strains with wild-type *PAH1* and promoter replacement showed no significant difference in OD_600_. The p*HXT1*-*PAH1* and p*REV1*-*PAH1* modifications did not significantly improve ABA titers. However, the strain carrying the p*minCYC1*-*PAH1* modification, yMO39, produced 15.8 mg/L ABA, ≈1.3-fold the titer produced by the wild-type *PAH1* control yMO23. For all strains with modified *PAH1* promoters, the ABA titer normalized to OD_600_ remained at *PAH1* wild-type levels, which is about 40% lower than for the *pah1*Δ strain yMO26 (Fig. 5A).

In addition, we compared the growth profile and maximum growth rate μ_max_ of the background strain SCIGS22a, the wild-type *PAH1* strain yMO23, the *pah1*Δ strain yMO26 and the p*minCYC1*-*PAH1* strain yMO39 (Fig. 5B). The growth profiles and μ_max_ of yMO23 and yMO39 were similar, both had a lower μ_max_ and slightly lower final OD_600_ than the background strain. Among the analysed strains, the *pah1*Δ strain yMO26 had the most severe growth defect in terms of μ_max_ and final OD_600_. Interestingly, yMO26 did not show bi-phasic growth. Deletion of *PAH1* has been reported to cause respiratory deficiencies [92], potentially explaining the growth defect.

In conclusion, by exchanging p*PAH1* for the weak minimal promoter p*minCYC1*, we were able to avoid the growth defect observed in the *pah1*Δ strain yMO26, while still improving ABA titers. The relative ABA titer per OD_600_ was higher for yMO26 than for yMO39, indicating that ABA production might be further improved by fine-tuning ER proliferation. We assume that p*PAH1* replacement could be a promising engineering strategy in *Y. lipolytica* ABA cell factories.

### Expression of *A. thaliana* proteins to improve ABA production

The *A. thaliana* proteins *At*MSBP1, *At*COL4 and *At*GRP7 were previously found to significantly improve CYP activity in a betaxanthin-producing *S. cerevisiae* strain [39]. Motivated by this, we sought to investigate the proteins’ effects on ABA production. However, difficulties were encountered during the chemical synthesis of the *AtGRP7* gene. *At*GPR7 also only had minor effects on CYP activity in previous work [39] and we therefore decided to exclude the protein from our study. *AtMSBP1* and *AtCOL4* were individually expressed by integrating a single copy of each gene into the ySMC001 genome (a strain based on SABA3 with single copies of each *bcaba* gene and *bccpr1*), controlled by strong constitutive promoters p*TEF2* for *AtMSBP1* and p*HHF2* for *AtCOL4*, resulting in the strains ySMC023 and ySMC024, respectively. The strain overexpressing *AtMSBP1* produced 16.6 mg/L ABA, a 3.5-fold increased ABA titer compared to ySMC001 (Fig. 6). While expression of *AtCOL4* alone did not influence ABA production, co-expression of both, *AtMSBP1* and *AtCOL4*, in strain ySMC025 significantly increased the titer to 26 mg/L, a 5.5-fold increase compared to the starting strain ySMC001 and a 1.6-fold increase to ySMC023 carrying solely *AtMSBP1*. The relative ABA titer for ySMC023 and ySMC025 was also improved compared to ySMC001, however no significant difference between ySMC023 and ySMC025 was observed (Fig. 6). Expression of the *A. thaliana* genes did not affect OD_600_.

To further improve ABA production, we added the *AtMSBP1* cassette alone or in combination with *AtCOL4* to the previously best performing strain yMO39, resulting in strains ySMC027 and ySMC028. Besides the *A. thaliana* genes, these strains also carry additional copies of *bcaba1* and *bcaba2*, express *bccyb5* and *bccbr1* and include the *pPAH1Δ::pminCYC1* modification. Expression of *AtMSBP1* in strain ySMC027 did not alter OD_600_, nor did it affect absolute or normalized ABA titer compared to yMO39 (Fig. 6). Co-expression of *AtMSBP1* and *AtCOL4* in ySMC028 severely reduced OD_600_ while no beneficial effect was visible for the absolute ABA titer. However, when normalized to the OD_600_, the ABA titer was increased in this strain.

Finally, we investigated whether further enhancing the MVA pathway flux could benefit ABA production. To achieve this, we expressed an additional copy of *ERG20* under the control of p*TEF2* in the strains ySMC025 and ySMC028 resulting in strains ySMC026 and ySMC029, respectively. No significant increase in absolute ABA titers was achieved when compared to the respective parental strains (Fig. 6). While the addition of an extra *ERG20* gene in the strain ySMC026 did not result in a higher ABA/OD_600_ ratio, in the ySMC029 strain, the additional copy resulted in the highest ABA/OD_600_ ratio of 9.3 (Fig. 6). These results support the idea that adding an extra copy of *ERG20* directs the pathway flux toward product formation. The effect is particularly important in strains derived from the yMO39 background; however, the OD_600_ was severely impaired in this strain, likely due to the metabolic burden caused by extensive genetic engineering.

Strikingly, the titers of ABA achieved by the highly engineered strain ySMC027 (21.90 mg/L) were comparable to those obtained in the less modified strain ySMC025 (26.22 mg/L), in which only a single copy of the P450s, *bcaba1* and *bcaba2*, are present (Fig. 6). The expression of the *Arabidopsis* genes seems to be the most straightforward and effective strategy, as it requires few engineering steps and does not adversely affect the OD_600_.

We speculate that the enhancement in ABA production observed with *AtMSBP1* expression is likely due to its dual role as a chaperone-like protein and a structural scaffold for the assembly of ER-bound enzymes [40, 93–95]. Subcellular localization studies showed that MSBP1 from *Saponaria vaccaria* co-localizes with CYPs on the ER membrane of yeast [93, 94], suggesting a potential interaction that could be leveraged for enhanced ABA biosynthesis. The boost in ABA production observed when *AtCOL4* is co-expressed with *AtMSBP1*, but not for *AtCOL4* alone, suggests that the two *Arabidopsis* proteins interact. While *At*COL4 is known to enhance abiotic stress tolerance and regulate ABA biosynthesis genes in *Arabidopsis* [96], its molecular mechanisms in yeast remains unclear. It is worth noting that the ABA biosynthetic pathway differs substantially in plants and fungi [10] and the effect of *At*COL4 is not specific for enzymes involved in ABA production [39]. In ySMC027 the higher expression levels of *bcaba1* and *bcaba2*, the presence of *bccyb5* and *bccbr1*, and the *PAH1-*modulation-mediated ER alterations might result in non-optimal assembly of enzyme complexes (i.e. due to different stoichiometries), potentially interfering with the *At*MSBP1- and *At*COL4-mediated effect. Further studies will be required to elucidate the underlying mechanism.

The hypothesis that enzyme complexes on the ER-membrane can improve enzyme activity is also supported by a recent study by Sun et al. [20]. The authors found that linking BcABA1 and BcABA2 via short peptide tags improved ABA production in *Y. lipolytica* by 38%. This beneficial effect appears to be enzyme-specific since linking BcABA3 and an ERG20 mutant with the same peptide tags did not increase ABA titers [93].

During the revision of this paper, a new study reported that expression of *AtMSBP1* broadly enhanced plant CYP performance in yeast [97], providing an additional example of its effectiveness. Importantly, the authors observed that At*MSBP1* expression induces not only ER expansion but also causes an increase in mitochondrial volume and inducing vacuole fission [97] . Furthermore, overexpression of the genes encoding proteins involved in organelle coordination such as *RTN1* (reticulon 1), *VMA2* (vacuolar ATPase subunit), *AFG1* (ATPase family gene 1), *MERG3* (mitochondrial genome regulator) and *PHB1* (prohibitin 1) enhanced CYP efficiency*.* Interestingly, co-expressing *AtMSBP1* with these proteins did not result in an additive effect on 1-OH-N-methylcanadine production, suggesting redundancy in their roles as CYP-supporting proteins. This is similar to the apparent redundancy we observed when combining *AtMSBP1* expression with the *PAH1* knockdown. Nonetheless, our findings open new avenues for designing and fine-tuning strategies that integrate *AtMSBP1* and *AtCOL4* expression with additional organelle-remodeling targets to optimize yeast cells factories for ABA production.

## Conclusion

The microbial production of ABA through engineered cell factories offers a promising and sustainable alternative to other methods such as extraction from plants or chemical synthesis. While the ABA biosynthetic pathway can be successfully reconstituted in *S. cerevisiae*, optimizing its performance remains challenging, particularly due to the complexity of CYP enzyme activity in relation to the cellular environment. We investigated various strategies to enhance ABA production in yeast, including modulation of transporter expression, adding additional CYP gene copies, expressing CYB5, improving heme and oxygen supply, ER proliferation and heterologous expression of *A. thaliana* genes. Surprisingly, we found that co-expression of the *Arabidopsis* genes *AtMSBP1* and *AtCOL4* improved ABA titers several fold. Combining the most promising engineering strategies, namely additional CYP gene copies, ER proliferation and *Arabidopsis* gene expression did not result in further improvements. This and other recent studies show that optimizing CYP functionality in cell factories is complex and its underlying principles are still not completely understood. In this regard, our findings provide valuable insights not just for heterologous ABA biosynthesis but also for potential CYP-expression platform strains.

## Supplementary Information

Additional file 1.

## Data Availability

All data generated and/or analysed during this study are included in this published article and the additional information file.

## References

[1] Finkelstein R. Abscisic acid synthesis and response. *The Arabidopsis Book*. Vol 11. 2013. 10.1199/tab.0166 PMC3833200 24273463

[2] Ortiz-García P, González Ortega-Villaizán A, Onejeme FC, et al. Do opposites attract? Auxin-abscisic acid crosstalk: new perspectives. Int J Mol Sci. 2023;24:3090. 10.3390/ijms24043090 PMC9960826 36834499

[3] Gupta K, Wani SH, Razzaq A, et al. Abscisic acid: role in fruit development and ripening. Front Plant Sci. 2022;10;13:817500. 10.3389/fpls.2022.817500 PMC9127668 35620694

[4] Olds CL, Glennon E, Luckhart S. Abscisic acid: new perspectives on an ancient universal stress signaling molecule. Microbes Infect. 2018;20:484–92. 10.1016/j.micinf.2018.01.009 29408537

[5] Kim SW, Goossens A, Libert C, et al. Phytohormones: multifunctional nutraceuticals against metabolic syndrome and comorbid diseases. Biochem Pharmacol. 2020;175:113866. 10.1016/j.bcp.2020.113866 32088261

[6] Lievens L, Pollier J, Goossens A, et al. Abscisic acid as pathogen effector and immune regulator. Front Plant Sci. 2017;8:00587. 10.3389/fpls.2017.00587 PMC5395610 28469630

[7] Sakthivel P, Sharma N, Klahn P, et al. Abscisic acid: a phytohormone and mammalian cytokine as novel pharmacon with potential for future development into clinical applications. Curr Med Chem. 2016;23:1549–70. 27048335 10.2174/0929867323666160405113129

[8] Pydi SP, Jaggupilli A, Nelson KM, et al. Abscisic acid acts as a blocker of the bitter taste G protein-coupled receptor T2R4. Biochemistry. 2015;54:2622–31. 25844797 10.1021/acs.biochem.5b00265

[9] Shi T-Q, Peng H, Zeng S-Y, et al. Microbial production of plant hormones: opportunities and challenges. Bioengineered. 2016;8:124–8. 10.1080/21655979.2016.1212138 PMC5398602 27459344

[10] Hirai N, Yoshida R, Todoroki Y, et al. Biosynthesis of abscisic acid by the non-mevalonate pathway in plants, and by the mevalonate pathway in fungi. Biosci Biotechnol Biochem. 2014;64:1448–58. 10.1271/bbb.64.1448 10945263

[11] Inomata M, Hirai N, Yoshida R, et al. The biosynthetic pathway to abscisic acid via ionylideneethane in the fungus *Botrytis cinerea*. Phytochemistry. 2004;65:2667–78. 15464154 10.1016/j.phytochem.2004.08.025

[12] Siewers V, Smedsgaard J, Tudzynski P. The P450 monooxygenase BcABA1 is essential for abscisic acid biosynthesis in *Botrytis cinerea*. Appl Environ Microbiol. 2004;70. 10.1128/AEM.70.7.3868-3876.2004 PMC444755 15240257

[13] Siewers V, Kokkelink L, Smedsgaard J, et al. Identification of an abscisic acid gene cluster in the grey mold *Botrytis cinerea*. Appl Environ Microbiol. 2006;72:4619–26. 16820452 10.1128/AEM.02919-05 PMC1489360

[14] Takino J, Kozaki T, Ozaki T, et al. Elucidation of biosynthetic pathway of a plant hormone abscisic acid in phytopathogenic fungi. Biosci Biotechnol Biochem. 2019;83:1642–9. 31112101 10.1080/09168451.2019.1618700

[15] Otto M, Teixeira PG, Vizcaino MI, et al. Integration of a multi-step heterologous pathway in *Saccharomyces cerevisiae* for the production of abscisic acid. Microb Cell Fact. 2019;18:205. 31767000 10.1186/s12934-019-1257-z PMC6876084

[16] Meadows AL, Hawkins KM, Tsegaye Y, et al. Rewriting yeast central carbon metabolism for industrial isoprenoid production. Nature. 2016;537:694. 27654918 10.1038/nature19769

[17] Paddon CJ, Westfall PJ, Pitera DJ, et al. High-level semi-synthetic production of the potent antimalarial artemisinin. Nature. 2013;496:528–32. 23575629 10.1038/nature12051

[18] Arnesen JA, Jacobsen IH, Dyekjær JD, et al. Production of abscisic acid in the oleaginous yeast *Yarrowia lipolytica*. FEMS Yeast Res. 2022;22:foac015. 35274684 10.1093/femsyr/foac015 PMC8992728

[19] Song X, Zhang J, Wang X, et al. Enhancement of abscisic acid biosynthesis in *Saccharomyces cerevisiae* via multidimensional engineering. Process Biochem. 2024;146:515–24.

[20] Sun M-L, Zou Z, Lin L, et al. Systematic metabolic engineering of *Yarrowia lipolytica* for efficient production of phytohormone abscisic acid. Synth Syst Biotechnol. 2025;10:165–73. 39552760 10.1016/j.synbio.2024.10.004 PMC11564786

[21] Denisov IG, Makris TM, Sligar SG, et al. Structure and chemistry of cytochrome P450. Chem Rev. 2005;105:2253–78. 15941214 10.1021/cr0307143

[22] Jiang L, Huang L, Cai J, et al. Functional expression of eukaryotic cytochrome P450s in yeast. Biotechnol Bioeng. 2021;118:1050–65. 33205834 10.1002/bit.27630

[23] Podust LM, Sherman DH. Diversity of P450 enzymes in the biosynthesis of natural products. Nat Prod Rep. 2012;29:1251–66. 22820933 10.1039/c2np20020a PMC3454455

[24] Werck-Reichhart D, Feyereisen R. Cytochromes P450: a success story. Genome Biol. 2000;1:reviews3003.1. 11178272 10.1186/gb-2000-1-6-reviews3003 PMC138896

[25] Forman V, Luo D, Kampranis SC, et al. Not all cytochrome b5s are created equal: how a specific CytB5 boosts forskolin biosynthesis in *Saccharomyces cerevisiae*. Metab Eng. 2024;86:288–99. 39454871 10.1016/j.ymben.2024.10.008

[26] Zhang H, Im S-C, Waskell L. Cytochrome b5 increases the rate of product formation by Cytochrome P450 2B4 and competes with Cytochrome P450 reductase for a binding site on Cytochrome P450 2B4. J Biol Chem. 2007;282:29766–76. 17693640 10.1074/jbc.M703845200

[27] Durairaj P, Li S. Functional expression and regulation of eukaryotic cytochrome P450 enzymes in surrogate microbial cell factories. Eng Microbiol. 2022;2:100011. 39628612 10.1016/j.engmic.2022.100011 PMC11610987

[28] Arendt P, Miettinen K, Pollier J, et al. An endoplasmic reticulum-engineered yeast platform for overproduction of triterpenoids. Metab Eng. 2017;40:165–75. 28216107 10.1016/j.ymben.2017.02.007

[29] Kim J-E, Jang I-S, Son S-H, et al. Tailoring the *Saccharomyces cerevisiae* endoplasmic reticulum for functional assembly of terpene synthesis pathway. Metab Eng. 2019;56:50–9. 31445083 10.1016/j.ymben.2019.08.013

[30] Pascual F, Carman GM. Phosphatidate phosphatase, a key regulator of lipid homeostasis. Biochim Biophys Acta. 2013;1831:514–22. 22910056 10.1016/j.bbalip.2012.08.006 PMC3549317

[31] Henry SA, Kohlwein SD, Carman GM. Metabolism and regulation of glycerolipids in the yeast *Saccharomyces cerevisiae*. Genetics. 2012;190:317–49. 22345606 10.1534/genetics.111.130286 PMC3276621

[32] Schuck S, Prinz WA, Thorn KS, et al. Membrane expansion alleviates endoplasmic reticulum stress independently of the unfolded protein response. J Cell Biol. 2009;187:525–36. 19948500 10.1083/jcb.200907074 PMC2779237

[33] Michener JK, Nielsen J, Smolke CD. Identification and treatment of heme depletion attributed to overexpression of a lineage of evolved P450 monooxygenases. Proc Natl Acad Sci U S A. 2012;109:19504–9. 23129650 10.1073/pnas.1212287109 PMC3511110

[34] Savitskaya J, Protzko RJ, Li F-Z, et al. Iterative screening methodology enables isolation of strains with improved properties for a FACS-based screen and increased L-DOPA production. Sci Rep. 2019;9:5815. 30967567 10.1038/s41598-019-41759-0 PMC6456618

[35] Ishchuk OP, Domenzain I, Sánchez BJ, et al. Genome-scale modeling drives 70-fold improvement of intracellular heme production in *Saccharomyces cerevisiae*. Proc Natl Acad Sci U S A. 2022;119:e2108245119. 35858410 10.1073/pnas.2108245119 PMC9335255

[36] Lin J-Y, Bu X, Lan Y-B, et al. Combined metabolic engineering and lipid droplets degradation to increase vitamin A production in *Saccharomyces cerevisiae*. Microb Cell Fact. 2024;23:317. 39581972 10.1186/s12934-024-02596-7 PMC11587636

[37] Mirończuk AM, Kosiorowska KE, Biegalska A, et al. Heterologous overexpression of bacterial hemoglobin VHb improves erythritol biosynthesis by yeast *Yarrowia lipolytica*. Microb Cell Fact. 2019;18:176. 31615519 10.1186/s12934-019-1231-9 PMC6794898

[38] Wei X-X, Chen G-Q. Chapter Fifteen - Applications of the VHb gene *vgb* for improved microbial fermentation processes. In: Poole RK, editor. Methods in enzymology, vol. 436. Academic Press; 2008. p. 273–87. 10.1016/S0076-6879(08)36015-7 18237638

[39] Jiang L, Dong C, Liu T, et al. Improved functional expression of cytochrome P450s in *Saccharomyces cerevisiae* through screening a cDNA library from *Arabidopsis thaliana*. Front Bioeng Biotechnol. 2021;9:764851. 10.3389/fbioe.2021.764851 PMC8696027 34957066

[40] Gou M, Ran X, Martin DW, et al. The scaffold proteins of lignin biosynthetic cytochrome P450 enzymes. Nat Plants. 2018;4:299–310. 29725099 10.1038/s41477-018-0142-9

[41] Lyu H-N, Fu C, Chai X, et al. Systematic thermal analysis of the *Arabidopsis* proteome: thermal tolerance, organization, and evolution. Cell Syst. 2023;14:883-894.e4. 37734376 10.1016/j.cels.2023.08.003

[42] Steinbach Y. The *Arabidopsis thaliana* CONSTANS-LIKE 4 (COL4) – a modulator of flowering time. Front Plant Sci. 2019;57:313–24. 10.3389/fpls.2019.00651 PMC6546890 31191575

[43] Sukarta OCA, Zheng Q, Slootweg EJ, et al. GLYCINE-RICH RNA-BINDING PROTEIN 7 potentiates effector-triggered immunity through an RNA recognition motif. Plant Physiol. 2022;189:972–87. 35218353 10.1093/plphys/kiac081 PMC9157115

[44] Slovik S, Baier M, Hartung W. Compartmental distribution and redistribution of abscisic acid in intact leaves : i. Mathematical formulation. Plant 1992;187:1–25. 24177962 10.1007/BF00201619

[45] Heilmann B, Hartung W, Gimmler H. The distribution of abscisic acid between chloroplasts and cytoplasm of leaf cells and the permeability of the chloroplast envelope for abscisic acid. Z Pflanzenphysiol. 1980;97:67–78.

[46] Kaiser WM, Hartung W. Uptake and release of abscisic acid by isolated photoautotrophic mesophyll cells, dependin on pH gradients. Plant Physiol. 1981;68:202–6. 16661871 10.1104/pp.68.1.202 PMC425916

[47] Mira NP, Teixeira MC, Sá-Correia I. Adaptive response and tolerance to weak acids in *Saccharomyces cerevisiae*: a genome-wide view. OMICS. 2010;14:525–40. 20955006 10.1089/omi.2010.0072 PMC3129613

[48] Kolaczkowska A, Goffeau A. Regulation of pleiotropic drug resistance in yeast. Drug Resistance Updates Rev Commentaries Antimicrob Anticancer Chemother. 1999;2:403–14. 10.1054/drup.1999.0113 11498356

[49] Le Crom S, Devaux F, Marc P, et al. New insights into the pleiotropic drug resistance network from genome-wide characterization of the *YRR1* transcription factor regulation system. Mol Cell Biol. 2002;22:2642–9. 11909958 10.1128/MCB.22.8.2642-2649.2002 PMC133742

[50] Mumberg D, Müller R, Funk M. Yeast vectors for the controlled expression of heterologous proteins in different genetic backgrounds. Gene. 1995;156:119–22. 7737504 10.1016/0378-1119(95)00037-7

[51] Merksamer PI, Trusina A, Papa FR. Real-time redox measurements during endoplasmic reticulum stress reveal interlinked protein folding functions. Cell. 2008;135:933–47. 10.1016/j.cell.2008.10.011 PMC2739138 19026441

[52] Chen X, Li X, Ji B, et al. Suppressors of amyloid-β toxicity improve recombinant protein production in yeast by reducing oxidative stress and tuning cellular metabolism. Metab Eng. 2022;72:311–24. 35508267 10.1016/j.ymben.2022.04.005

[53] Jessop-Fabre MM, Jakočiūnas T, Stovicek V, et al. EasyClone-MarkerFree: a vector toolkit for marker-less integration of genes into *Saccharomyces cerevisiae* via CRISPR-Cas9. Biotechnol J. 2016;11:1110–7. 27166612 10.1002/biot.201600147 PMC5094547

[54] Mans R, Rossum HMvan, Wijsman M, et al. CRISPR/Cas9: a molecular Swiss army knife for simultaneous introduction of multiple genetic modifications in *Saccharomyces cerevisiae*. FEMS Yeast Res. 2015;15:fov004. 25743786 10.1093/femsyr/fov004 PMC4399441

[55] Engler C, Kandzia R, Marillonnet S. A one pot, one step, precision cloning method with high throughput capability. PLoS ONE. 2008;3:e3647. 18985154 10.1371/journal.pone.0003647 PMC2574415

[56] Lee ME, DeLoache WC, Cervantes B, et al. A highly characterized yeast toolkit for modular, multipart assembly. ACS Synthetic Biol. 2015;4:975–86. 10.1021/sb500366v 25871405

[57] Otto M, Skrekas C, Gossing M, et al. Expansion of the yeast modular cloning toolkit for CRISPR-based applications, genomic integrations and combinatorial libraries. ACS Synth Biol. 2021;10:3461–74. 34860007 10.1021/acssynbio.1c00408 PMC8689691

[58] Verduyn C, Postma E, Scheffers WA, et al. Effect of benzoic acid on metabolic fluxes in yeasts: a continuous-culture study on the regulation of respiration and alcoholic fermentation. Yeast. 1992;8:501–17. 1523884 10.1002/yea.320080703

[59] van Dijken JP, Bauer J, Brambilla L, et al. An interlaboratory comparison of physiological and genetic properties of four *Saccharomyces cerevisiae* strains. Enzyme Microb Technol. 2000;26:706–14. 10862876 10.1016/s0141-0229(00)00162-9

[60] López J, Essus K, Kim I, et al. Production of β-ionone by combined expression of carotenogenic and plant CCD1 genes in *Saccharomyces cerevisiae*. Microb Cell Fact. 2015;14:84. 26063466 10.1186/s12934-015-0273-x PMC4464609

[61] Gietz RD, Woods RA. Yeast transformation by the LiAc/SS carrier DNA/PEG method. In: Xiao W, editor. Yeast protocol. Totowa, NJ: Humana Press; 2006. p. 107–20. 10.1385/1-59259-958-3:107 16118429

[62] Shaw WM, Yamauchi H, Mead J, et al. Engineering a model cell for rational tuning of GPCR signaling. Cell. 2019;177:782-796.e27. 10.1016/j.cell.2019.02.023 PMC6476273 30955892

[63] Novarina D, Koutsoumpa A, Milias-Argeitis A. A user-friendly and streamlined protocol for CRISPR/Cas9 genome editing in budding yeast. STAR Protoc. 2022;3:101358. 35712010 10.1016/j.xpro.2022.101358 PMC9192979

[64] RStudio Team. RStudio: Integrated Development for R. 2022.

[65] Wickham H, Averick M, Bryan J, et al. Welcome to the tidyverse. J Open Source Softw. 2019;4:1686.

[66] Lenth R, Piaskowski J. emmeans: Estimated Marginal Means, aka Least-Squares Means.

[67] Hothorn T, Bretz F, Westfall P. multcomp - simultaneous inference in general parametric models. 10.1002/bimj.200810425 18481363

[68] Petzoldt T. R package growthrates. 2022.

[69] Ro D-K, Ouellet M, Paradise EM, et al. Induction of multiple pleiotropic drug resistance genes in yeast engineered to produce an increased level of anti-malarial drug precursor, artemisinic acid. BMC Biotechnol. 2008;8:83. 18983675 10.1186/1472-6750-8-83 PMC2588579

[70] Buechel ER, Pinkett HW. Transcription factors and ABC transporters: from pleiotropic drug resistance to cellular in yeast. FEBS Lett. 2020;594:3943–64. 33089887 10.1002/1873-3468.13964

[71] Sun M-L, Xu Y, Lin L, et al. Enhancing precursor supply and engineering efflux systems to improve abscisic acid production and secretion in *Yarrowia lipolytica*. J Agric Food Chem. 2025;73:6050–8. 40011064 10.1021/acs.jafc.4c10772

[72] Wang G, Møller-Hansen I, Babaei M, et al. Transportome-wide engineering of *Saccharomyces cerevisiae*. Metab Eng. 2021;64:52–63. 33465478 10.1016/j.ymben.2021.01.007 PMC7970624

[73] Scalcinati G, Partow S, Siewers V, et al. Combined metabolic engineering of precursor and co-factor supply to increase α-santalene production by *Saccharomyces cerevisiae*. Microb Cell Fact. 2012;11:1–16. 22938570 10.1186/1475-2859-11-117 PMC3527295

[74] Zhang Z, Moo-Young M, Chisti Y. Plasmid stability in recombinant *Saccharomyces cerevisiae*. Biotechnol Adv. 1996;14:401–35. 14540156 10.1016/s0734-9750(96)00033-x

[75] Fang F, Salmon K, Shen MWY, et al. A vector set for systematic metabolic engineering in *Saccharomyces cerevisiae*. Yeast. 2011;28:123–36. 20936606 10.1002/yea.1824 PMC3070743

[76] Singh MV, Anthony Weil P. A method for plasmid purification directly from yeast. Anal Biochem. 2002;307:13–7. 12137773 10.1016/s0003-2697(02)00018-0

[77] Schenkman JB, Jansson I. The many roles of cytochrome b5. Pharmacol Ther. 2003;97:139–52. 12559387 10.1016/s0163-7258(02)00327-3

[78] Han G-S, Wu W-I, Carman GM. The *Saccharomyces cerevisiae* lipin homolog is a Mg2+-dependent phosphatidate phosphatase enzyme. J Biol Chem. 2006;281:9210–8. 16467296 10.1074/jbc.M600425200 PMC1424669

[79] Kudo S, Shiino H, Furuta S, et al. Yeast transformation stress, together with loss of Pah1, phosphatidic acid phosphatase, leads to Ty1 retrotransposon insertion into the *INO4* gene. FASEB J. 2020;34:4749–63. 32037626 10.1096/fj.201901811RR

[80] Zhang X, Liu X, Meng Y, et al. Combinatorial engineering of *Saccharomyces cerevisiae* for improving limonene production. Biochem Eng J. 2021;176:108155.

[81] Heyken W-T, Repenning A, Kumme J, et al. Constitutive expression of yeast phospholipid biosynthetic genes by variants of Ino2 activator defective for interaction with Opi1 repressor. Mol Microbiol. 2005;56:696–707. 15819625 10.1111/j.1365-2958.2004.04499.x

[82] Nguyen PTM, Ishiwata-Kimata Y, Kimata Y. Fast-Growing *Saccharomyces**cerevisiae* cells with a constitutive unfolded protein response and their potential for lipidic molecule production. Appl Environ Microbiol. 2022;88:e01083-e1122. 10.1128/aem.01083-22 PMC9642017 36255243

[83] Papagiannidis D, Bircham PW, Lüchtenborg C, et al. Ice2 promotes ER membrane biogenesis in yeast by inhibiting the conserved lipin phosphatase complex. EMBO J. 2021;40:e107958. 34617598 10.15252/embj.2021107958 PMC8591542

[84] Qu Z, Zhang L, Zhu S, et al. Overexpression of the transcription factor *HAC1* improves nerolidol production in engineered yeast. Enzyme Microb Technol. 2020;134:109485. 32044032 10.1016/j.enzmictec.2019.109485

[85] Liu Q, Liu Y, Li G, et al. De novo biosynthesis of bioactive isoflavonoids by engineered yeast cell factories. Nat Commun. 2021;12:6085. 34667183 10.1038/s41467-021-26361-1 PMC8526750

[86] Faulkner A, Chen X, Rush J, et al. The *LPP1* and *DPP1* gene products account for most of the isoprenoid phosphate phosphatase activities in *Saccharomyces cerevisiae*. J Biol Chem. 1999;274:14831–7. 10329682 10.1074/jbc.274.21.14831

[87] Carman GM. Discoveries of the phosphatidate phosphatase genes in yeast. J Biol Chem. 2019;294:1681–9. 30061152 10.1074/jbc.TM118.004159 PMC6364789

[88] Ottoz DSM, Rudolf F, Stelling J. Inducible, tightly regulated and growth condition-independent transcription factor in *Saccharomyces cerevisiae*. Nucleic Acids Res. 2014;42:e130–e130. 25034689 10.1093/nar/gku616 PMC4176152

[89] Scalcinati G, Knuf C, Partow S, et al. Dynamic control of gene expression in *Saccharomyces cerevisiae* engineered for the production of plant sesquitepene α-santalene in a fed-batch mode. Metab Eng. 2012;14:91–103. 22330799 10.1016/j.ymben.2012.01.007

[90] Skjoedt ML, Snoek T, Kildegaard KR, et al. Engineering prokaryotic transcriptional activators as metabolite biosensors in yeast. Nat Chem Biol. 2016;12:951–8. 10.1038/nchembio.2177 27642864

[91] Hahn S, Hoar ET, Guarente L. Each of three “TATA elements” specifies a subset of the transcription initiation sites at the promoter of *Saccharomyces cerevisiae*. Proc Natl Acad Sci USA. 1985;82:8562–6. 3001709 10.1073/pnas.82.24.8562 PMC390957

[92] Irie K, Takase M, Araki H, et al. A gene, *SMP2*, involved in plasmid maintenance and respiration in *Saccharomyces cerevisiae* encodes a highly charged protein. Mol Gen Genet. 1993;236:283–8. 8437575 10.1007/BF00277124

[93] Liu L, Zhao K, Liu Z. Construction and regulation of the abscisic acid biosynthesis pathway in *Yarrowia lipolytica*. J Agric Food Chem. 2024;72:7299–307. 38504621 10.1021/acs.jafc.4c00223

[94] Liu Y, Zhao X, Gan F, et al. Complete biosynthesis of QS-21 in engineered yeast. Nature. 2024;629:937–44. 38720067 10.1038/s41586-024-07345-9 PMC11111400

[95] Yang J, Liu Y, Zhong D, et al. Combinatorial optimization and spatial remodeling of CYPs to control product profile. Metab Eng. 2023;80:119–29. 37703999 10.1016/j.ymben.2023.09.004 PMC10698227

[96] Min J-H, Chung J-S, Lee K-H, et al. The CONSTANS-like 4 transcription factor, *At*COL4, positively regulates abiotic stress tolerance through an abscisic acid-dependent manner in *Arabidopsis*. J Integr Plant Biol. 2015;57:313–24. 25073793 10.1111/jipb.12246

[97] Xu S, Wei S, Xiong Y, et al. Enhancing cross-organelle coordination to advance plant cytochrome P450 functionality in yeast. Sci Adv. 2025;24;11:eady7184. 10.1126/sciadv.ady7184 PMC12551693 41134884

