# Supplementary material for: Engineering Saccharomyces cerevisiae to improve heterologous abscisic acid production
Source: Microb Cell Fact. 2026 Jan 23;25:24. doi: 10.1186/s12934-025-02913-8 (PMC12836960; doi:10.1186/s12934-025-02913-8)
Supplement: Supplementary file 1 — Additional file 1. [file 12934_2025_2913_MOESM1_ESM.pdf]

Supplementary Material: Engineering *Saccharomyces cerevisiae* to Improve Heterologous Abscisic Acid Production

Maximilian Otto [1†], Sara Muñiz-Calvo [1†], Michael Gossing [1, 2], Florian David [1], Verena Siewers [1, 3 *]

[1] Division of Systems and Synthetic Biology, Department of Life Sciences, Chalmers University of Technology, Gothenburg, Sweden

[2] Discovery Sciences, Biopharmaceuticals R&D, AstraZeneca, Gothenburg, Sweden

[3] Novo Nordisk Foundation Center for Biosustainability, Technical University of Denmark, Kgs. Lyngby, Denmark

[†] Authors contributed equally

∗Corresponding author. Department of Life Sciences, Chalmers University of Technology, 412 96 Gothenburg, Sweden. E-mail: siewers@chalmers.se

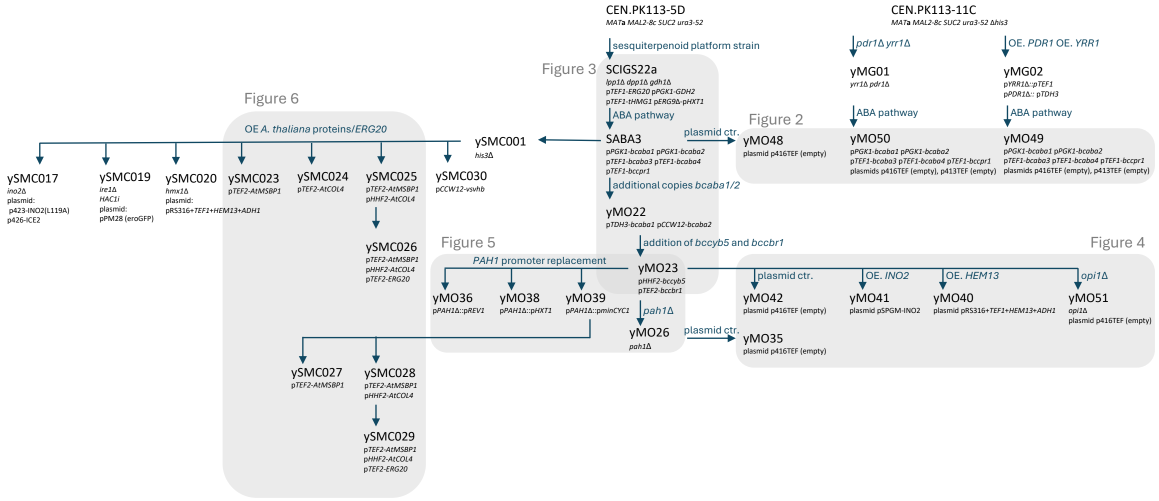

Figure S1. Strain pedigree tree of parent strains and strains constructed in this study. Grey boxes indicate strains that were compared in Figures 2-6. For more detailed strain information see Table 1 in Materials and Methods section. OE = overexpression.

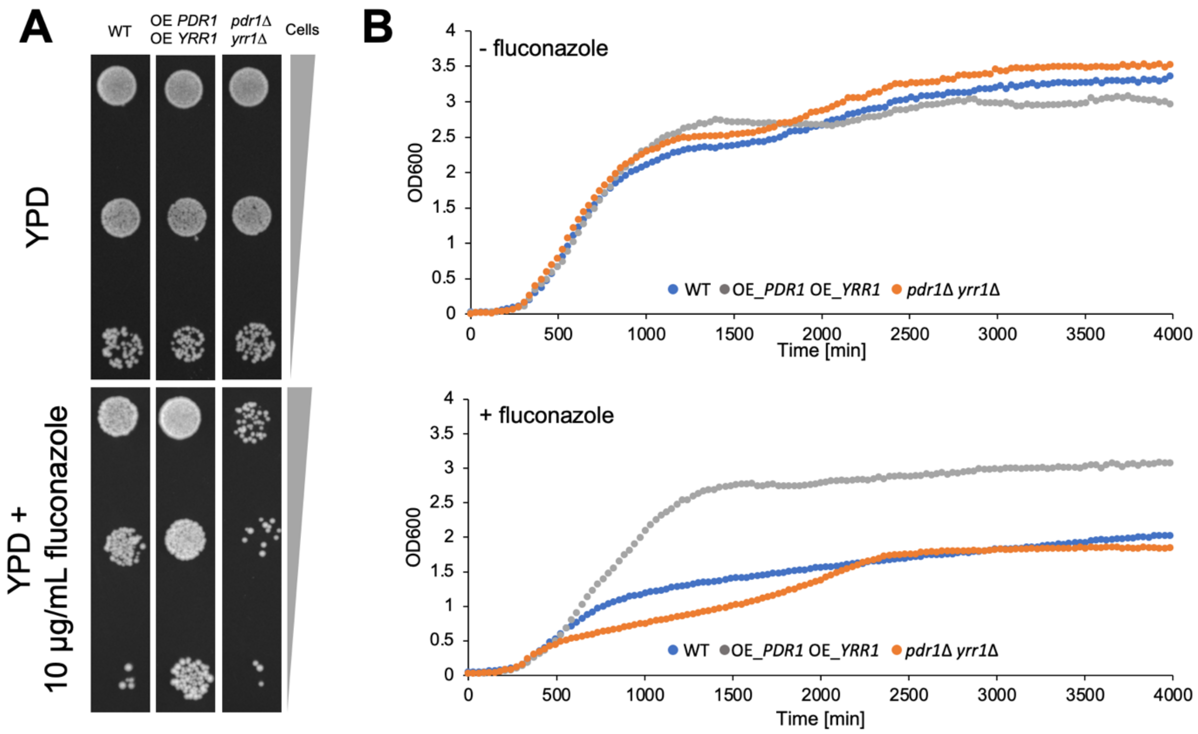

Figure S2: Effect of fluconazole on *PDR1+YRR1* overexpression (OE) or knock-out strains. **A** Cells were grown to mid log phase (OD≈0.5) in YPD, and diluted to OD=0.05, OD=0.01 and OD=0.001. 5 µL of these dilutions were spotted onto YPD and YPD+10 µg/mL fluconazole agar plates and were cultivated for 3 d at 30 °C. WT, CEN.PK113-11C; *pdr1Δ yrr1Δ*, yMG01; OE *PDR1* OE *YRR1*, yMG02. **B** Cells were cultivated in 250 µL SD (top panel) and SD+15 µg/mL fluconazole (bottom panel) at 30 °C, in a 96-well microtiter plate. Starting OD=0.01. Growth was monitored in an Enzyscreen Growth Profiler 960. Data points are the mean of technical triplicates.

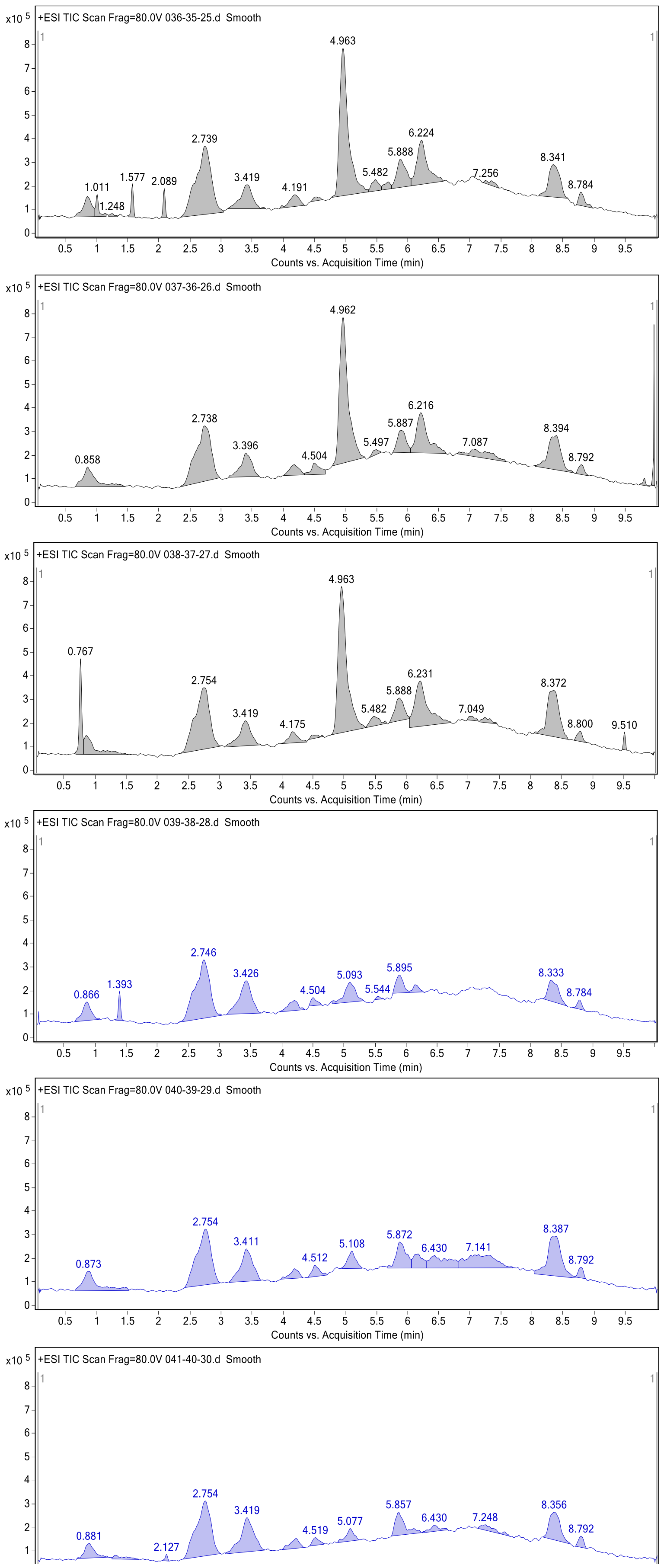

Figure S3: Total ion chromatograms of supernatant of yMO48 (grey, wild-type *PDR1* and *YRR1*) and yMO49 (blue, overexpression of *PDR1* and *YRR1*), 3 replicates are shown per strain

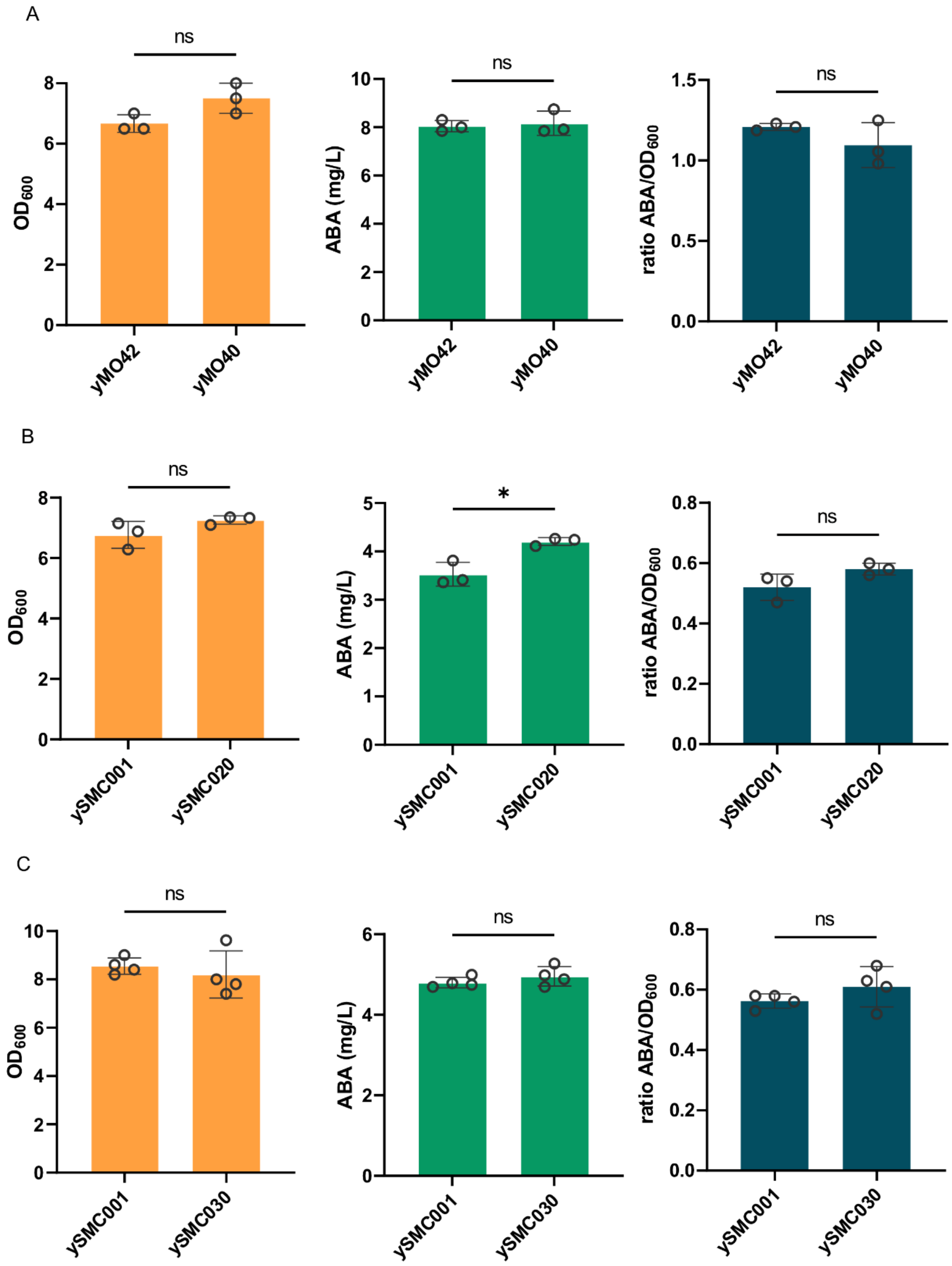

Figure S4: Effect of modulation of heme metabolism and oxygen supply on different ABA-producing strains. (A) Effect of *HEM13* episomal overexpression (yMO40) and the control strain yMO42 (yMO23 carrying an empty plasmid). Strains were cultivated for 60 h in minimal medium (24-deepwell microplates). (B) Effect of the combination of *HEM13* overexpression and *HMX1* deletion (ySMC020) and the control strain ySMC001. Strains were cultivated for 72 h in minimal media supplemented with 60 mg/L of uracil and histidine. (C) Effect of *vsvhb* overexpression (ySMC030) and the control strain ySMC001. Strains were cultivated for 48 h in minimal media supplemented with 100 mg/L of uracil and histidine OD_{600} (orange), ABA titer in the supernatant (green) and ABA titer normalized to OD_{600} (blue). Grey circles show the individual data points used to calculate the mean and standard deviation. The statistical analyses were performed using unpaired two-tailed Student's t-test. *$P < 0.05$ ns, not significant ($p > 0.05$)

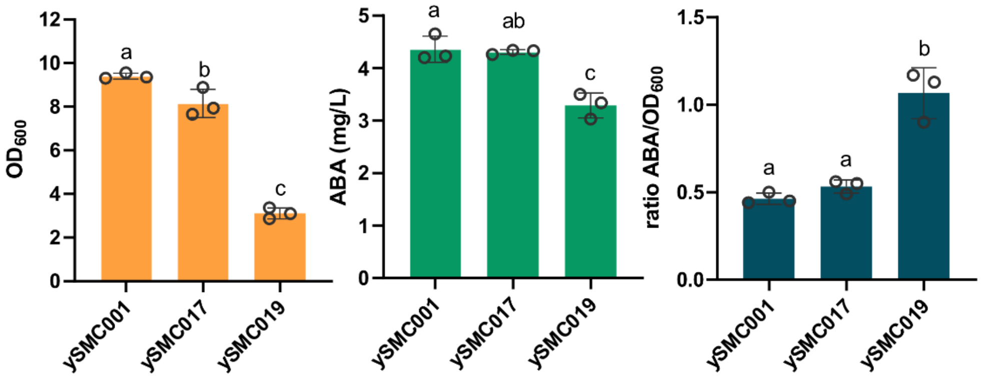

Figure S5: Effect of engineering ER membrane biogenesis and UPR response on different ABA-producing strains. OD_{600} (orange), ABA titer in the supernatant (green) and ABA titer normalized to OD_{600} (blue) are shown after 72 h of cultivation in mineral media (24- deepwell microplates) supplemented with 100 mg/L of uracil and histidine. The *INO2* gene was deleted in control strain ySM001 (*INO2*, *IRE1*, and *HAC1* WT) and then transformed with episomal plasmids carrying *ICE2* and *INO2* (L119A) (ySMC017). Alternatively, *IRE1* was deleted in control strain ySM001, and the eroGFP expression plasmid pPM28 together with an inducible form of *HAC1* (*HAC1i*) was introduced (ySMC019). More detailed information about the strains' genotypes can be found in Table 1. Grey circles show the individual data points used to calculate the mean and standard deviation. To study the significance of the differences between strains, a one-way ANOVA was used followed by Tukey's test ($\alpha = 0.05$). Shared letters indicate no significant difference.

Table S1: Primers used in this study

Primer name	Sequence
**306**	GCATCGTCTCATCGGTCTCATATGTCTAACTCAATCTTGAATTTGGG
**307**	ATGCCGTCTCAGGTCTCAGGATCCTTATTTGTATTCTGTACCTTCAG
**308**	GCATCGTCTCATCGGTCTCATATGTTGTTATCTATTAAAGATTTG
**309**	ATGCCGTCTCAGGTCTCAGGATCCTTATCTTGGAACTTCTTTTAAC
**310**	TATCAGAATGAGGCCACCATTGTTGGGT
**357**	ACCCAACAATGGTGGCCTCATTCTGATA
**366**	GAAGAGTAGGCCATCGTGGG
**367**	CAGCAAGCGAGTTTCGTGTG
**417**	GGACAAGTGTTCAGAGCAGTATAATGTTGCTTCTGTATCAAATGACCCTAACCGTAGTCGGTCTCAAACG
**418**	GAAGACCATGTTTTAGACACAGACCCAAGAGCTCTGCCTACGTACTGCATAGATCTCGCTGGATATGCC
**422**	GGACAAGTGTTCAGAGCAGTATAATGTTGCTTCTGTATCAAATGACCCTAGCAGGTCCCATCTGGAATATAATTCC
**423**	GAAGACCATGTTTTAGACACAGACCCAAGAGCTCTGCCTACGTACTGCATGATTTTACGTATATCAACTAGTTGACGATTATG
**424**	GGACAAGTGTTCAGAGCAGTATAATGTTGCTTCTGTATCAAATGACCCTACTAGAGCATGTGCTCTGTATGTATA
**425**	GAAGACCATGTTTTAGACACAGACCCAAGAGCTCTGCCTACGTACTGCATAAGCTTGATATCGAATTCCTGC
**426**	CCAACTCGATTTCGCTCAGC
**427**	GCTCTGCCTACGTACTGCAT
**1260**	TCCACCTCAATGAGAGGGATTAA
**pTDH3-PDR1 ampl fwd**	TCGAGTTTATCATTATCAATACTGC
**pTDH3-PDR1 ampl rev**	TTTGTTTGTTTATGTGTGTTTATTCG
**pTEF1-YRR1 ampl fwd**	GCACACACCATAGCTTCAAAATG

**pTEF1-YRR1 ampl rev**	TTTGTAATTAAAACTTAGATTAGATTGCTATG
**PDR1 fwd**	CAGCCACTCTTTCATTGTCAAC
**PDR1 rev**	CGTTGAGGATCAAATGAATATCC
**YRR1 fwd**	CATTTATAGTATTCCACATCACTAGACG
**YRR1 rev**	CGGTAAGCAGCGATTCAGC
**F-HR fwd**	GAAGCTTCAGCTGACG
**R-HR rev**	GCTGCAGGTCGACAAC
**1261**	GGTCCTTACTGAGCCATTCAAAGG
**oSMC1**	GACTTTAGTTGTCTCCTTCCAGTTCG
**oSMC2**	AAACCGAACTGGAAGGAGACAACTAA
**oSMC3**	AGCATGGGTTTTGATCAAACGCTTGGTCTCAAGTTGTCTCCTTCCAGTTCAGGATTGGCG
**oSMC4**	GTCTAAAAACTGTTCAATGGCATTCGATTCATTCGTGCTCGCCAATCCTGAACTGGAAGG
**oSMC5**	AATTCGAAATTGGTTCGGTTC
**oSMC6**	TCGCCCATGTATACGAAATT
**oSMC11**	AAGCGTTCCAATGAAACTCT
**oSMC14**	ACACCAATAACTCTTCCTAT
**oSMC15**	GCATCGTCTCATCGGTCTCATATGGAAATGACTGATTTTGAAC
**oSMC16**	CTGGATTACGCCAATTGTCA
**oSMC17**	TGACAATTGGCGTAATCCAGAAGCGCAGTCAGGTTTGAAT
**oSMC18**	ATGCCGTCTCAGGTCTCAGGATCCTCATGAAGTGATGAAGAAATCATTC
**oSMC25**	GCCCGGGATGACCAGTTTGTCCAAAAG
**oSMC26**	CCGCTCGAGTCAACTACCAGAACCTATTAA
**oSMC27**	GCCCGGGATGCAACAAGCAACTGGGAA
**oSMC28**	CCGCTCGAGTCAGGAATCATCCAGTATGTGC
**oSMC31**	ATGGAAATGACTGATTTTGAAC
**oSMC32**	TCATGAAGTGATGAAGAAATCATTC
**oSMC33**	GACTTTACCTTCAAAAGCAGAATGCA
**oSMC34**	AAACTGCATTCTGCTTTTGAAGGTAA
**oSMC35**	GCATCGTCTCATCGGTCTCAAACGTGTGGA
**oSMC36**	ATGCCGTCTCAGGTCTCAAGAATCTCACATAGATCTTATTTTATTGTATTG
**oSMC37**	GCATCGTCTCATCGGTCTCAAACGTTGAT
**oSMC38**	ATGCCGTCTCAGGTCTCAAGAAGTTTAGTTAATTATAGTTCGTTGAC
**oSMC39**	GCATCGTCTCATCGGTCTCAAACGCACCC
**oSMC40**	ATGCCGTCTCAGGTCTCAAGAATATTGATATAGTGTTTAAGCGAATGACAGA
**oSMC45**	CCTGCAGGACTAGTGCTGAG
**oSMC57**	GACTTTATTGCGATCTCTTTAAAGGG
**oSMC58**	AAACCCCTTTAAAGAGATCGCAATAA
**oSMC59**	ACCCGTTGCCATGTGCATA
**oSMC60**	ACAAGGATCACTGGCACAGT
**oSMC67**	GAGAACGAGAGGACCCAACAT
**oSMC78**	CGGTAGGTATTGATTGTAATTCTG
**oSMC84**	GCGTGAATGTAAGCGTGAC
**oSM89**	ATGCCTCGGTAATGATTTTC
**oSM90**	ATACCACTTGCCACCTATCA

**oSMC123**	GCATCGTCTCATCGGTCTCATATGGCTTCAGAAAAAGAAATTAG
**oSMC124**	ATGCCGTCTCAGGTCTCAGGATCCTTATTTACTTCTCTTGTAAACCTTG

Table S2: Standard PCR reactions with annealing temperature T_{ann} and elongation time t_{elo} (in min:sec)

Primers	Template	Polymerase	T_{ann}	t_{elo}	Description
**292/293**	*bccyb5* (Genscript)	Phusion	53°C	0:30	*bccyb5* attach MoClo overhangs
**294/295**	*bccbr1* (Genscript)	Phusion	53°C	0:30	*bccbr1* attach MoClo overhangs
**306/357**	p416-ABA1 (Otto *et al.* 2019)	PrimeStar	55°C	1:15	*bcaba1* remove BsaI site and attach MoClo overhangs
**307/310**	p416-ABA1 (Otto *et al.* 2019)	PrimeStar	55°C	1:15	*bcaba1* remove BsaI site and attach overhangs
**308/309**	p416-ABA2 (Otto *et al.* 2019)	PrimeStar	55°C	2:15	*bcaba2* attach MoClo overhangs
**366/367**	*S. cerevisiae* genomic DNA	SapphireAmp	55°C	0:35	validation Δ*pah1*
**417/418**	pYTK027 (Lee *et al.* 2015)	PrimeStar	55°C	1:30	repair cassette p*REV1*
**422/423**	*S. cerevisiae* genomic DNA	PrimeStar	55°C	1:30	repair cassette p*HXT1*
**424/425**	FRP793_insul-(lexA-box)4-PminCYC1-Citrine-TCYC1 (Ottoz, Rudolf and Stelling 2014)	PrimeStar	55°C	0:20	repair cassette p*minCYC1*
**426/427**	*S. cerevisiae* genomic DNA	SapphireAmp	55°C	0:20	validation p*PAH1*
**1260/1261**	*S. cerevisiae* genomic DNA	SapphireAmp	50°C	0:20	validation Δ*opi1*
**oSMC11/oSMC14**	*S. cerevisiae* genomic DNA	SapphireAmp	49.5°C	0:10	validation Δ*ire1*
**oSMC89/oSMC90**	*S. cerevisiae* genomic DNA	SapphireAmp	52.5°C	0:10	validation Δ*his3*
**oSMC5/oSMC6**	*S. cerevisiae* genomic DNA	SapphireAmp	53°C	0:10	validation Δ*ino2*
**oSMC25/ oSMC26**	*S. cerevisiae* genomic DNA	Phusion	56.9°C	1:15	*ICE2* attach RE sites XmaI/XhoI for cloning
**oSMC27/ oSMC28**	*S. cerevisiae* genomic DNA	Phusion	63°C	1:00	*INO2* attach RE sites XmaI/XhoI for cloning
**oSMC59/oSMC60**	*S. cerevisiae* genomic DNA	SapphireAmp	57.5°C	0:10	validation Δ*hmx1*
**oSMC35/ oSMC36**	*S. cerevisiae* genomic DNA	Phusion	59.4°C	0:30	p*HHF2* attach MoClo overhangs
**oSMC37/ oSMC38**	*S. cerevisiae* genomic DNA	Phusion	57.4°C	0:30	p*TEF2* attach MoClo overhangs
**oSMC39/ oSMC40**	*S. cerevisiae* genomic DNA	Phusion	63.9°C	0:30	p*CCW12* attach MoClo overhangs
**oSMC123/oSMC124**	*S. cerevisiae* genomic DNA	Phusion	58.5°C	0:45	*ERG20* attach MoClo overhangs
**HIS3 UP KO/ HIS3 DOWNKO**	no template PCR	Phusion	63.6°C	0:20	Donor DNA used as repair fragment for *HIS3* deletion
**IRE1 KO UP/ IRE1 KO DOWN**	no template PCR	Phusion	55.8°C	0:20	Donor DNA used as repair fragment for *IRE1* deletion
**INO2 KO UP/ INO2 KO DOWN**	no template PCR	Phusion	57.7°C	0:20	Donor DNA used as repair fragment for *INO2* deletion

**HMX1 KO UP/ HMX1 KO DOWN**	no template PCR	Phusion	57.6°C	0:20	Donor DNA used as repair fragment for *HMX1* deletion
**oSMC3/oSMC4**	no template PCR	Phusion	66°C	0:20	Donor DNA used as repair fragment for the introduction of the point mutation L119A into *INO2*

Table S3: List of MoClo assemblies. Protocol according to Lee *et al.* (2015) and Otto *et al.* (2021). All plasmids with the "pYTK" prefix originate from the Lee et al. paper (2015).

Plasmid name	MoClo parts used	MoClo level	Note
**pMC3-bcaba1**	pYTK001<br>PCR product 306/307	0	
**pMC3-bcaba2**	pYTK001<br>PCR product 308/309	0	
**pMC3-bccyb5**	pYTK001<br>PCR product 292/293	0	
**pMC3-bccbr1**	pYTK001<br>PCR product 294/295	0	
**pMC-Ura-Cen**	pYTK002<br>pYTK047<br>pYTK067<br>pYTK074<br>pYTK081<br>pYTK081	1 (backbone)	contains GFP dropout
**pMC-His-Cen**	pYTK003<br>pYTK047<br>pYTK072<br>pYTK076<br>pYTK081<br>pYTK083	1 (backbone)	contains GFP dropout
**pMMC16**	pMC-Ura-Cen<br>pYTK009<br>pMC3-bcaba1<br>pYTK056	1	
**pMMC17**	pMC-His single<br>pYTK010<br>pMC3-bcaba2<br>pYTK055	1	
**pMMC24**	pMC-Ura single<br>pYTk12<br>pMC3-bccyb5<br>pYTK054	1	
**pMMC25**	pMC-His single<br>pYTK14<br>pMC3-bccbr1<br>pYTK052	1	
**pX3-bcaba1+2**	pMC-X3<br>pMMC16<br>pMMC17	2	
**pXII2-bccyb5+cbr1**	pMC-XII2<br>pMMC24<br>pMMC25	2	
**pSMC-atcol4**	pYTK003<br>PCR product oSMC35/ oSMC36<br>Plasmid ordered with atcol4<br>pYTK054<br>pYTK072<br>pYTK083	1	
**pSMC-atmsbp1**	pYTK002<br>PCR product oSMC37/ oSMC38<br>Plasmid ordered with atmsbp1<br>pYTK055<br>pYTK067<br>pYTK074	1	

	pYTK082<br>pYTK083		
**pSMC-vsvhb**	pYTK002<br>PCR product<br>oSMC39/ oSMC40<br>Plasmid ordered<br>with VHb<br>pYTK056<br>pYTK072<br>pYTK076<br>pYTK082<br>pYTK083	1	
**pX2-atcol4**	pMC-X2<br>pSMC-atcol4	2	
**pX2-msbp1**	pMC-X2<br>PSMC-atmsbp1	2	
**pX2-atcol4/atmsbp1**	pMC-X2<br>pSMC-atcol4<br>PSMC-atmsbp1	2	
**pXI2-vsvHb**	pMC-XI2<br>pSMC-vsvHb	2	
**pXI5-ERG20**	PMC-XI5<br>pYTK014<br>PCR product<br>oSMC123/oSMC124<br>pYTK056	2	

Table S4: Media composition

**YPD**	
yeast extract (Merck)	10 g/L
peptone from meat (Merck)	20 g/L
glucose (Merck)	20 g/L
for plates: agar agar (Merck)	20 g/L
**SD (pH adjusted to 6 with KOH)**	
complete supplement mix dropout -uracil (Formedium)	0.77 g/L
yeast-nitrogen base without amino acids (Formedium)	6.9 g/L
glucose (Merck)	20 g/L
for plates: agar agar (Merck)	20 g/L
**LB (pH adjusted to 6.5 with NaOH)**	
peptone from casein (Merck)	10 g/L
NaCl (Merck)	10 g/L
yeast extract (Merck)	5 g/L
for plates: agar agar (Merck)	20 g/L
**Mineral media (pH adjusted to 6.5 with KOH)**	
ammonium sulfate (Merck)	7.5 g/L
monopotassium phsophate (Merck)	14.4 g/L
magnesium sulfate heptahydrate (Merck)	0.5 g/L
glucose (Merck)	20 g/L
trace metal solution	2 mL/L
vitamin solution	1 mL/L
for plates: agar agar (Merck)	20 g/L
for auxotrophic strains: uracil/histidine(Alfa Aesar)	100 mg/L
**Trace metal solution**	
$FeSO_4 \bullet 7H_2O$	3 g/L
$ZnSO_4 \bullet 7H_2O$	4.5 g/L
$CaCl_2 \bullet 2H_2O$	4.5 g/L
$MnCl_2 \bullet 4H_2O$	1 g/L
$CoCl_2 \bullet 6H_2O$	300 mg/L
$CuSO_4 \bullet 5H_2O$	300 mg/L
$Na_2MoO_4 \bullet 2H_2O$	400 mg/L
H_3BO_3	1 g/L
KI	100 mg/L
$Na_2EDTA \bullet 2H_20$	19 g/L
**Vitamin solution**	
D-Biotin	50 mg/L
D-Pantothenic acid hemicalcium salt	1 g/L
thiamin-HCl	1 g/L
pyridoxin-HCl	1 g/L
nicotinic acid	1 g/L
4-aminobenzoic acid	0.2 g/L
myo-Inositol	25 g/L

Table S5: Oligonucleotides used in this study. gRNA target sequences are underlined.

Oligo description	Sequence
**pTDH3-PDR1 ampl fwd OL**	ATAGACATTACCAGGAGTGAGTTTTGGCTAAAAAAAGACAATTTGCACATCACTTGCTAATCGAGTTTATCATTATCAATACTGC
**pTDH3-PDR1 ampl rev OL v2**	CGATTCTGTATCCGGACCCGTCTCAATATGTACACCGTTCTTAGGTGTCAAGCCTCGCATTTTGTTTGTTTATGTGTGTTTATTCG
**pTEF1-YRR1 ampl fwd OL**	GCCAAAAATTCGGAATTACAAGAGAAAAGTTAGATCAAAGGAACAACAAGATCAACGAGAgcacacaccatagcttcaaaatg
**pTEF1-YRR1 ampl rev OL v2**	TGGCGGCGTAACGTTGGTGGCCTGGAAACTTCCCAACAAAGCATCGCTTCTTCTTTTCATtttgtaattaaaacttagattagattgctatg
**PDR1 repair fwd**	ATAGACATTACCAGGAGTGAGTTTTGGCTAAAAAAAGACAATTTGCACATCACTTGCTAAACGTATACGTTTGTATAGATAAAAGTTCTCAAAAACTTCCTTCCTTTTTTTTTTTCTTTTG
**PDR1 repair rev**	CAAAAGAAAAAAAAAAAGGAAGGAAGTTTTTGAGAACTTTTATCTATACAAACGTATACGTTTAGCAAGTGATGTGCAAATTGTCTTTTTTTAGCCAAAACTCACTCCTGGTAATGTCTAT
**YRR1 repair fwd**	GCCAAAAATTCGGAATTACAAGAGAAAAGTTAGATCAAAGGAACAACAAGATCAACGAGATGCTAAGTCGAGGCAATCTCGCGGCGCGACTACTTTGAAGCCTTTTTTTAGTTCTTTTTT
**YRR1 repair rev**	AAAAAAGAACTAAAAAAAGGCTTCAAAGTAGTCGCGCCGCGAGATTGCCTCGACTTAGCATCTCGTTGATCTTGTTGTTCCTTTGATCTAACTTTTCTCTTGTAATTCCGAATTTTTGGC
**Repair oligo Δ*pah1***	TTTTACCTTCTAAGAAACATACAGGGAAGACATTACTGAAGATAGACACATCGGTCGATTAGATTCTTGTAGCCGAATATTATTTATAACGATCCATACTGCATATTAAGTAAAATTATG
**gRNA fragment Δ*pah1***	TGCGCATGTTTCGGCGTTCGAAACTTCTCCGCAGTGAAAGATAAATGATCTGATTGAGGGGGGCTTGATGGTTTTAGAGCTAGAAATAGCAAGTTAAAATAAGGCTAGTCCGTTATCAAC
**Repair oligo Δ*opi1***	TTAAAGCGTGTGTATCAGGACAGTGTTTTTAACGAAGATACTAGTCATTGCCTCTAATACATCCAACACTCTACGCCCTCTTCAAGAGCTAGAAGGGCACCCTGCAGTTGGAAAGGGAATTATTTCGTAAGGCGAGCCCATACCGTCATTCATGCGGAAGAGTTAACACGATTGGAAGTAGGAATAGTTTCGAACCACGGTTACTAATCCTAATAACGGAACGCTGTCTGAAGGATGAGTGTCAGCGAGTGTAACTCGATGAGCTACCCAGTAGTCGTACTGGTCGAGACAACATTGTACCCCAGCGGCGGCGCGGCCAGCTCTAATGCACTCAATCCCGAGGCCTGACGCGACATATCAGCTTAGACTAGGGCGGGGGTGTTGACGTTTGGGGTTGAATAAATCTATTGTACTAATCGGCTTCAACGTGCCCCACGGGTGGCACCTCAGGAGGGGCCCACAGCGAGGAAGTAAACTGTTATTCGTCGGCGATGGTGGTAGCTAATTATGTTCCTTGCCACTACAATAGTATCTAAGCCGTGTAATGGGAACATCCACACCCGAGACAGATTGAGGTCTTTCATGCATTACCACCAGTAATAATATTATA
**gRNA fragment Δ*opi1***	TGCGCATGTTTCGGCGTTCGAAACTTCTCCGCAGTGAAAGATAAATGATCTGTCGCGGGCGATTGCCAAGGTTTTAGAGCTAGAAATAGCAAGTTAAAATAAGGCTAGTCCGTTATCAAC
**gRNA fragment p*PAH1* replacement**	TGCGCATGTTTCGGCGTTCGAAACTTCTCCGCAGTGAAAGATAAATGATCTTAGAGAATGAGCAGCACGTGTTTTAGAGCTAGAAATAGCAAGTTAAAATAAGGCTAGTCCGTTATCAAC
**HIS3 KO UP**	AATGTGATTTCTTCGAAGAATATACTAAAAAATGAGCAGGCAAGATAAACGAAGGCAAAGTGACACCGAT
**HIS3 KO DOWN**	GGTATACATATATACACATGTATATATATCGTATGCTGCAGCTTTAAATAATCGGTGTCACTTTGCCTTC
**IRE1 KO UP**	CCTTCATACACATTAAAAAAACAGCATATCTGAGGAATTAATATTTTAGCACTTTGAAAACATGTTCATG
**IRE1 KO DOWN**	ATGATCAAAGTAACATTAATGCAATAATCAACCAAGAAGAAGCAGAGGGGCATGAACATGTTTTCAAAGT
**INO2 KO UP**	TGGTTCGGTTCCATCTCGTTGACGTGCAATAAATAAATACATGGAACAGCAAAGGAGAAAGCTCATACAA
**INO2 KO DOWN**	AAATTAACTAAAAACACATCCAACGGGAGGCCATTTTCATCACTAATAGCTTGTATGAGCTTTCTCCTTT
**HMX1 KO UP**	TTAACAGTGCACAATATAACACAGCATATATACACACACACACATAAAATAACCGCAAAACGTATAAAAA
**HMX1 KO DOWN**	ATATTTTGATATTATTTCATGTATATATTATGTTTGTATTTAGACTTTTTTTTTTATACGTTTTGCGGTT
**INO2 L119A F**	AGCATGGGTTTTGATCAAACGCTTGGTCTCAAGTTGTCTCCTTCCAGTTCAGGATTGGCG
**INO2 L119A R**	GTCTAAAAACTGTTCAATGGCATTCGATTCATTCGTGCTCGCCAATCCTGAACTGGAAGG

Table S6: Heterologous gene sequences codon-optimised for *S. cerevisiae* and sequence of the minimal *CYC1* promoter (p*minCYC1*).

Sequence name	Sequence
AtCOL4	ATGGCATCTAAGTTGTGTGATTCTTGTAAGTCAGCTACAGCTGCATTGTATTGTAGACCAGATG<br>CTGCATTTTTATGTTTGTCTTGTGATTCAAAGGTTCATGCTGCAAATAAGTTAGCATCTAGACAT<br>GCTAGAGTTTGGATGTGTGAAGTTTGTGAACAAGCACCAGCTCATGTTACATGTAAAGCTGAT<br>GCTGCAGCTTTGTGTGTTACTTGTGATAGAGATATCCATTCAGCAAATCCATTGGCTAGAAGAC<br>ATGAAAGAGTTCCAGTTACACCATTTTATGATTCTGTTTCTTCAGATGGTTCAGTTAAGCATACT<br>GCTGTTAATTTCTTGGATGATTGTTACTTCTCTGATATCGATGGTAATGGTTCAAGAGAAGAAG<br>AAGAAGAAGAAGCAGCTTCTTGGTTGTTATTGCCAAATCCAAAAACTACAACTACAGCAACTGC<br>TGGTATTGTTGCAGTTACATCTGCTGAAGAAGTTCCTGGTGACTCACCAGAAATGAACACAGGT<br>CAACAATATTTGTTTTCTGATCCAGATCCATATTTGGATTTGGATTACGGTAATGTTGATCCAAA<br>GGTTGAATCATTGGAACAAAATTCTTCAGGTACTGATGGTGTTGTTCCAGTTGAAAACAGAACT<br>GTTAGAATACCAACAGTTAACGAAAACTGTTTCGAAATGGATTTCACTGGTGGTTCTAAAGGTT<br>TTACATATGGTGGTGGTTACAACTGTATCTCTCATTCAGTTTCTTCATCTTCAATGGAAGTTGGT<br>GTTGTTCCAGATGGTGGTTCTGTTGCAGATGTTTCATATCCATACGGTGGTCCAGCAACTTCTG<br>GTGCTGATCCAGGTACACAAAGAGCTGTTCCATTAACTTCAGCAGAAAGAGAAGCTAGAGTTA<br>TGAGATACAGAGAAAAGAGAAAGAATAGAAAGTTCGAAAAGACTATCAGATACGCATCAAGA<br>AAAGCATACGCTGAAATGAGGCCAAGAATTAAAGGTAGATTCGCTAAGAGAACTGATACAAAC<br>GAATCTAACGATGTTGTTGGTCATGGTGGTATTTTCTCTGGTTTTGGTTTGGTTCCAACTTTTTA<br>A
AtMSBP1	ATGGCTTTGGAATTGTGGCAAACATTAAAAGAAGCTATCCATGCTTACACTGGTTTGTCTCCTGT<br>CGTTTTCTTCACCGCTTTAGCTTTGGCTTTCGCTATTTACCAAGTTATCTCCGGTTGGTTCGCTTC<br>TCCATTCGACGATGTCAACAGACACCAAAGAGCCCGTTCATTGGCTCAAGAAGAAGAACCACC<br>AATTCCACAACCAGTTCAAGTCGGTGAAATTACTGAAGAAGAGTTAAAGCAATATGACGGCTCT<br>GACCCACAAAAGCCATTGTTGATGGCCATCAAGCACCAAATCTACGATGTTACTCAATCTAGAA<br>TGTTCTACGGTCCAGGTGGTCCATACGCCTTGTTTGCCGGTAAAGATGCCTCCAGAGCTCTAGC<br>CAAGATGTCTTTCGAAGAAAAGGACTTGACCTGGGACGTTTCTGGTTTGGGTCCTTTTGAATTG<br>GATGCTTTGCAAGACTGGGAATACAAGTTCATGTCCAAGTACGCTAAGGTTGGTACTGTCAAG<br>GTCGCTGGTAGTGAACCAGAAACTGCTTCCGTTTCCGAACCAACCGAAAACGTTGAACAAGAT<br>GCTCACGTTACCACCACTCCAGGTAAGACTGTTGTTGACAAGTCTGATGACGCTCCAGCTGAAA<br>CCGTCTTGAAGAAGGAAGAATGA
vsvhb	ATGTTGGACCAACAAACCATTAATATCATCAAAGCCACTGTTCCAGTTTTGAAGGAACACGGTG<br>TTACCATCACTACCACTTTCTATAAGAACCTTTTTGCTAAGCACCCAGAAGTTAGACCATTGTTC<br>GACATGGGTAGACAAGAATCTTTGGAACAACCAAAGGCTTTGGCTATGACTGTCTTGGCCGCC<br>GCCCAAAACATTGAAAACTTGCCAGCTATTTTACCTGCTGTTAAGAAGATCGCCGTCAAACATT<br>GTCAAGCTGGTGTTGCTGCCGCTCACTACCCAATTGTCGGTCAAGAATTGTTGGGTGCTATCAA<br>GGAAGTCCTAGGTGACGCTGCTACCGATGACATCTTGGATGCTTGGGGTAAGGCTTACGGTGT<br>CATTGCTGATGTTTTCATCCAAGTTGAAGCTGACTTATACGCTCAAGCTGTCGAATGA
**pminCYC1**	CTAGAGCATGTGCTCTGTATGTATATAAAACTCTTGTTTTCTTCTTTTCTCTTCTTTCCTTATACAT<br>TAGGACCTTTGCAGCATAAATTACTATACTTCTATACTAGTGGATCCCCCGGGCTGCAGGAATT<br>CGATATCAAGCTT

References

Otto M, Skrekas C, Gossing M *et al.* Expansion of the Yeast Modular Cloning Toolkit for CRISPR-Based Applications, Genomic Integrations and Combinatorial Libraries. *ACS Synthetic Biology* 2021;**10**:3461–74.

Otto M, Teixeira PG, Vizcaino MI *et al.* Integration of a multi-step heterologous pathway in *Saccharomyces cerevisiae* for the production of abscisic acid. *Microbial Cell Factories* 2019;**18**:205.

Ottoz DSM, Rudolf F, Stelling J. Inducible, tightly regulated and growth condition-independent transcription factor in *Saccharomyces cerevisiae*. *Nucleic Acids Research* 2014;**42**:e130–e130.
